# The genome and genetics of a high oxidative stress tolerant *Serratia* sp. LCN16 isolated from the plant parasitic nematode *Bursaphelenchus xylophilus*

**DOI:** 10.1186/s12864-016-2626-1

**Published:** 2016-04-23

**Authors:** Claudia S. L. Vicente, Francisco X. Nascimento, Yoriko Ikuyo, Peter J. A. Cock, Manuel Mota, Koichi Hasegawa

**Affiliations:** NemaLab/ICAAM - Instituto de Ciências Agrárias e Ambientais Mediterrânicas, Departamento de Biologia, Universidade de Évora, Núcleo da Mitra, Ap. 94, 7002-554, Évora, Portugal; Department of Environmental Biology, College of Bioscience & Biotechnology, Chubu University, 1200 Matsumoto, Kasugai, Aichi 487-8501 Japan; Information and Computational Sciences group (PJAC), The James Hutton Institute, Invergowrie, Dundee, DD2 5DA UK; Departamento de Ciências da Vida, Universidade Lusófona de Humanidades e Tecnologias, Lisboa, Portugal

**Keywords:** *Bursaphelenchus xylophilus*, Catalase, Endophyte, Reactive oxygen species, OxyR, *Serratia*, Oxidative stress, Pine wilt disease, Plant defenses

## Abstract

**Background:**

Pine wilt disease (PWD) is a worldwide threat to pine forests, and is caused by the pine wood nematode (PWN) *Bursaphelenchus xylophilus*. Bacteria are known to be associated with PWN and may have an important role in PWD. *Serratia* sp. LCN16 is a PWN-associated bacterium, highly resistant to oxidative stress in vitro, and which beneficially contributes to the PWN survival under these conditions. Oxidative stress is generated as a part of the basal defense mechanism used by plants to combat pathogenic invasion. Here, we studied the biology of *Serratia* sp. LCN16 through genome analyses, and further investigated, using reverse genetics, the role of two genes directly involved in the neutralization of H_2_O_2_, namely the H_2_O_2_ transcriptional factor *oxyR*; and the H_2_O_2_-targeting enzyme, catalase *katA*.

**Results:**

*Serratia* sp. LCN16 is phylogenetically most closely related to the phytosphere group of *Serratia,* which includes *S. proteamaculans*, *S. grimessi* and *S. liquefaciens*. Likewise, *Serratia* sp. LCN16 shares many features with endophytes (plant-associated bacteria), such as genes coding for plant polymer degrading enzymes, iron uptake/transport, siderophore and phytohormone synthesis, aromatic compound degradation and detoxification enzymes. OxyR and KatA are directly involved in the high tolerance to H_2_O_2_ of *Serratia* sp. LCN16. Under oxidative stress, *Serratia* sp. LCN16 expresses *katA* independently of OxyR in contrast with *katG* which is under positive regulation of OxyR. *Serratia* sp. LCN16 mutants for *oxyR* (*oxyR*::int(614)) and *katA* (*katA*::int(808)) were sensitive to H_2_O_2_ in relation with wild-type*,* and both failed to protect the PWN from H_2_O_2_-stress exposure. Moreover, both mutants showed different phenotypes in terms of biofilm production and swimming/swarming behaviors.

**Conclusions:**

This study provides new insights into the biology of PWN-associated bacteria *Serratia* sp. LCN16 and its extreme resistance to oxidative stress conditions, encouraging further research on the potential role of this bacterium in interaction with PWN *in planta* environment.

**Electronic supplementary material:**

The online version of this article (doi:10.1186/s12864-016-2626-1) contains supplementary material, which is available to authorized users.

## Background

The prevalence of pine wilt disease (PWD) in European and Asian forestlands causes significant environmental and economical effects, which have encouraged vulnerable countries to strengthen pest control management policies [1, 2]. The primary pathogenic agent of PWD is the plant-parasitic nematode *Bursaphelenchus xylophilus* (pine wood nematode, PWN) [3, 4]. PWN infects coniferous trees, mostly *Pinus* sp., using an insect-vector, *Monochamus* sp., for tree-to-tree transmission [5]. In the last decade, the parasitism of *B. xylophilus* has been intensively investigated [6–10]. In 2011, Kikuchi et al. [11] published a draft genome sequence for PWN revealing its distinct and unique parasitism tools including enzymes for metabolism of the host cell wall and detoxification enzymes. Shinya and co-workers [12] investigated the PWN secretome and identified a range of secreted cell-wall degrading enzymes and host-defense evasion proteins, among which 12 antioxidant enzymes (PRX, peroxiredoxin; CAT, catalase; GPX, glutathione peroxidase; nucleoredoxin-like proteins; SOD, superoxide dismutase; TRX, thioredoxin) were identified. More recently, Vicente et al. [13] showed the importance of PWN catalases in H_2_O_2_ detoxification in vitro, and Espada et al. [14] identified novel proteins involved in the host-parasite interaction and provided clear evidence that PWN employs a multilayered detoxification strategy to overcome plant defenses.

PWN-associated bacteria have been suggested to play an important role in the development of PWD (detailed review in Nascimento et al. [15]). A dual role has been attributed to these bacteria due to their phenotypic plasticity, expressing both plant pathogenic and plant growth promoting abilities [16]. These nematode-associated bacteria were initially seen as putative PWN’s symbiotic partners in PWD [17–19], though lately Paiva et al. [20] has shown also in vitro nematicidal activity of some associated bacteria. In spite of the intricate detoxification system present in PWN, Cheng et al. [21] and Vicente et al. [22] have shown the potential of PWN-associated bacteria, respectively, in the xenobiotic degradation and in the neutralization of H_2_O_2_.

Reactive oxygen species (ROS) have important roles in plant physiological processes such as growth and development, response to biotic and abiotic stresses and programmed cell death [23]. In host-pathogen interactions, apoplastic ROS production, also known as the oxidative burst, is one of the earliest detectable events in plant basal defenses [24]. This production is biphasic: the first phase is non-specific, relatively weak and occurs within minutes of the plant detecting a potential pathogen while the second occurs after prolonged pathogen attack, resulting in establishment of plant defenses and may be accompanied by a hypersensitive response [25]. H_2_O_2_, hydrogen peroxide, is the most stable, and membrane diffusible ROS [24]. H_2_O_2_ has a variety of roles in plants; at low concentrations it serves as a signaling molecule for the plant (e.g., in defense gene activation) but, at high concentrations, can lead to oxidative stress and cell death [26]. Avirulent pathogens induce biphasic ROS. However, in the case of virulent pathogens or symbiotic partners, which can avoid or suppress host recognition, only the first ROS wave is detected [27]. In these situations, both plant and pathogen attempt to regulate intracellular and extracellular ROS accumulation by employing several enzymatic and non-enzymatic antioxidants, such as: ascorbate peroxidases, GPXs, SODs, CAT or KAT, PRXs and glutathione S-transferases (GSTs) [28].

Vicente et al. [22] reported, for the first time, the high tolerance to oxidative stress of three PWN-associated bacteria (*Serratia* sp. LCN4, *Serratia* sp. LCN16, and *Serratia marcescens* PWN146), showing also the beneficial effect towards PWN under the same conditions. In the present work, we investigated the biology of *Serratia* sp. LCN16 through genome analyses, and further studied, using reverse genetics, the role of two genes directly involved in the neutralization of H_2_O_2_, namely the H_2_O_2_ transcriptional factor OxyR; and the H_2_O_2_ targeting enzyme, catalase (*katA*, hydroperoxidase II, HPII).

## Results

### Genome structure and general features

**Fig. 1:**
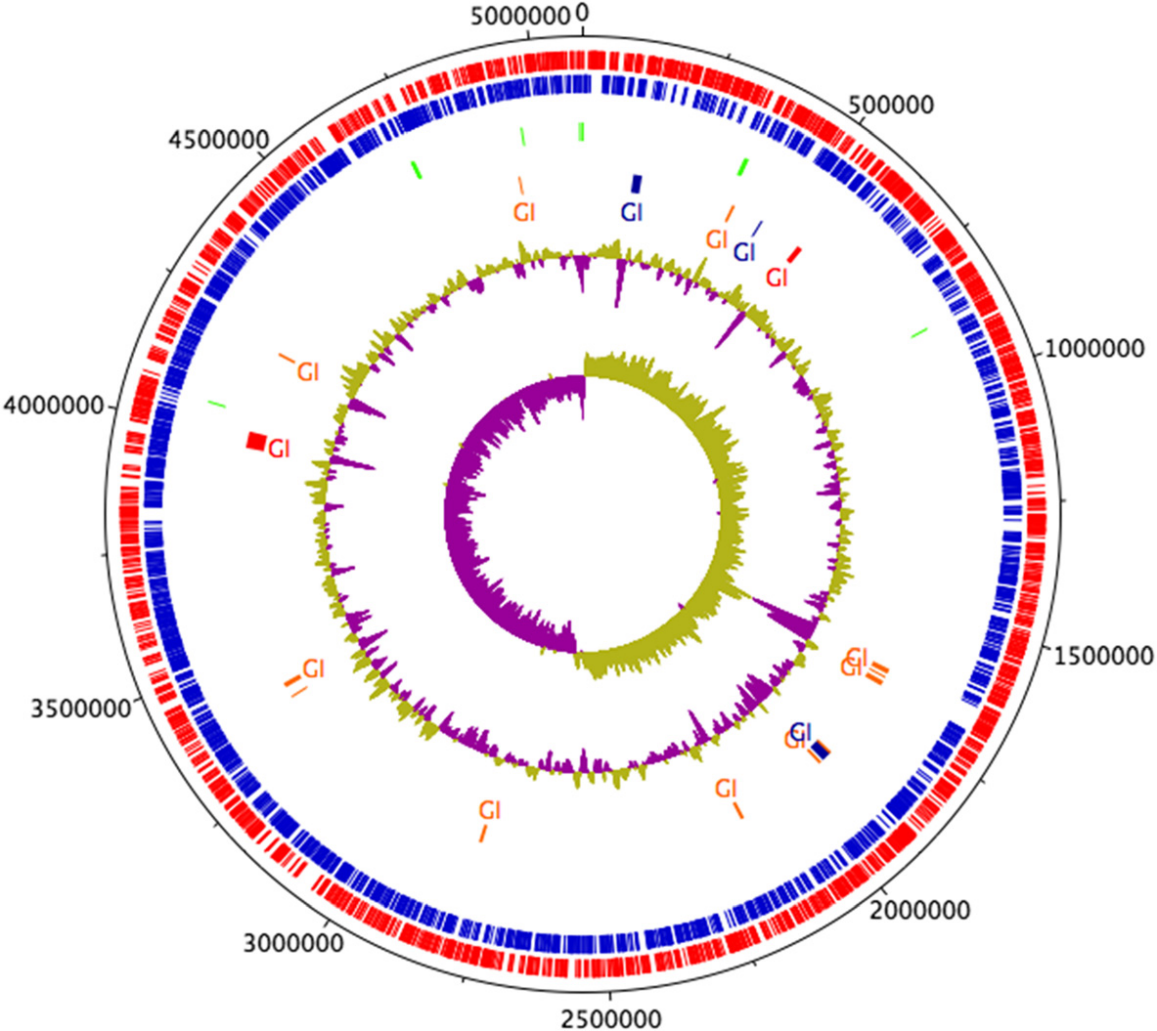
Circular representation of *Serratia* sp. LCN16. From the inner- to the outermost circle: circle 1, GC skew (positive GC skew in green and negative GC skew in purple); circle 2, GC plot; circle 3, predicted unique genomic regions of LCN16 known as genomic Islands (GI) [31]; circle 4, tRNA; circle 5, antisense strand (blue); and circle 6, sense strands (red). GIs in blue indicate prediction by IslandPath-DIMOB. GIs in orange indicate prediction following SIGI-HMM approach. GIs in red were predicted by both approaches

The draft genome of *Serratia* sp. LCN16 suggests a single chromosome of 5.09Mbp in size with an average GC content of 52.83 % (Fig. 1). Genome annotation predicts 4804 genes, of which 4708 were predicted protein coding sequences (CDS) and 96 were RNA genes (14 rRNA, 81 tRNA, and 1 tmRNA). Of the 4708 CDS, 4528 (96 %) were assigned InterPro entries, and 3413 (72 %) were assigned to Gene Ontology (GO) terms (Additional file 1: Table S1). Few mobile genetic elements (MGE) have been found to date: one transposase (LCN16_00783) and one transposon TN10 (LCN16_02368), and 35 putative phage sequences. No clustered regularly interspaced short palindromic repeats (CRISPR) were predicted in the *Serratia* sp. LCN16 genome.

**Fig. 2:**
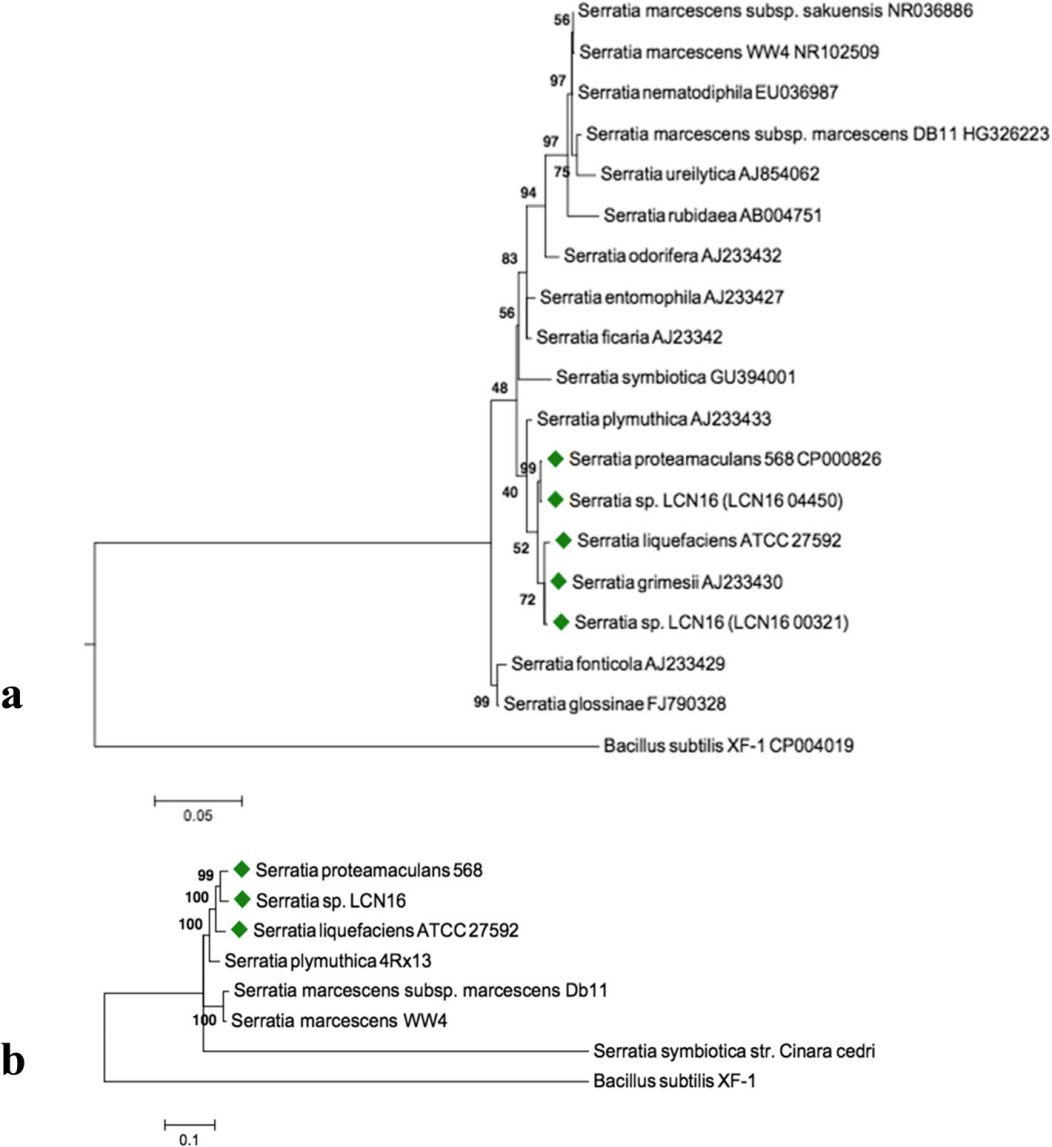
Phylogenetic relationships between *Serratia* sp. LCN16 and other *Serratia* representatives. Green diamonds indicate the phytosphere *Serratia* complex. **a** Phylogeny based on 16S rRNA gene (1411 bp). **b** Phylogeny based on housekeeping genes (*rpoB*, *gyrB*, *dnaJ* and *atpD*) (8808 bp). Numbers above the clades are bootstrap values (1,000 replicates). Maximum likelihood (ML) trees were constructed using: (A) GTR + G + I, generalized time-reversible model with gamma distribution and proportion of invariable sites; and (B) K2 + G, Kimura 2-parameters with gamma distribution. Model determination and construction of ML trees were performed in MEGA 6 [67]

A phylogenetic analysis of *Serratia* sp. LCN16 based on the 16S rRNA gene and four housekeeping genes (*rpoB*, *gyrB*, *dnaJ* and *atpD*) is shown in Fig. 2. Two copies of the 16S rRNA gene (LCN16_00312 and LCN16_04450), sharing 99.3 % similarity, were found in the *Serratia* sp. LCN16 genome. Both copies clustered within the phytosphere *Serratia* complex (*S. proteamaculans*, *S. grimessi* and *S. liquefaciens*) [29, 30], grouping with *S. proteamaculans* 568 (99 % bootstrap support), and *S. liquefaciens* ATCC 27592 and *S. grimessii* AJ233430 (72 % bootstrap). Furthermore, the phylogeny based on the housekeeping genes reinforces the clustering of *Serratia* sp. LCN16 with *S. proteamaculans* 568 (99 % bootstrap support) within *S. liquefaciens* ATCC 27592 clade (100 % bootstrap support). Based on these observations, the *Serratia* sp. LCN16 genome was aligned to the genomes of *S. proteamaculans* 568 and *S. liquefaciens* ATCC 27592. The three genomes are highly syntenic with few rearrangements (Additional file 2: Figure S1). *Serratia* sp. LCN16 and *S. liquefaciens* ATCC 27592 share a unique region of similarity which is not present in *S. proteamaculans* 568, and which encode hypothetical and phage proteins, an MsgA protein (DNA-damage-inducible protein I, DinI) and a peptidase P60 (a bacterial cell wall-degrading enzyme) (Additional file 2: Figure S1). A total of 19 genomic islands (GI) were identified by at least one of SIGI-HMM or IslandPath-DIMOB in IslandViewer server [31] (Fig. 1, Additional file 3: Table S2). The 8 GIs predicted by both methods [32] have a size range between 4 kb and 39 kb, and are rich in hypothetical proteins, phage elements and ABC transporters (i.e., lipopolyssacharides). Genes potentially involved in the synthesis of antimicrobials, such as *prnABD* for the biosynthesis of the antifungal antibiotic pyrrolnitrin (LCN16_00326-27; LCN16_00329), *kanB* (LCN16_03985) for kanamycin biosynthesis, and *mdtL* (LCN16_03987) which is involved in chloramphenicol resistance (Additional file 3: Table S2) are also present.

### *Serratia sp*. LCN16, a putative plant-associated bacterium

The phylogenetic analysis places *Serratia* sp. LCN16 within a phytosphere group of *Serratia* (Fig. 2) [29, 30]. Thus, to understand if *Serratia* sp. LCN16 is able to live in a plant-environment, a set of 40 genes predicted to be important for endophytic behavior of 11 plant-associated bacteria [33] was searched for in the genome (Table 1). *Serratia* sp. LCN16 has 34 of these genes (85 %), *S. proteamaculans* 568 has 37 genes (90 %) while *S. liquefaciens* ATCC 27592 has 33 genes (83 %). Other sequences were found in *Serratia* sp. LCN16, supporting the idea that they have the ability to colonize plants (Additional file 4: Table S3) [34, 35]. These include genes encoding proteins for plant polymer degradation, such as glycoside hydrolases (endoglucanase, LCN16_00161) and pectin (galacturonate) degradation (*uxaA* and *uxaC*, LCN16_04263-64), fungal chitin degradation as chitinases (LCN16_00148, LCN16_01150, LCN16_02767, LCN16_03549), complete pathways for degradation of aromatic compounds (KEGG: ko00362), such as benzoate or catechol degradation; and genes involved in the synthesis of plant growth regulating compounds, such as indole-pyruvate decarboxylase (*ipdC*, LCN16_00911 and LCN16_03478) for indole acetic acid (IAA); genes for acetoin and 2,3-butanediol production via *budABC* (LCN16_03505-6, LCN16_02073), and genes for polyamine synthesis (plant volatiles, putrescine and spermidine). Additionally, common among plant-associated bacteria is the ability for iron acquisition via siderophore synthesis or by iron uptake transporters and siderophore receptors [36]. *Serratia* sp. LCN16 encodes the complete pathway for siderophore biosynthesis (*entABEC*, LCN16_03491-94; *entF*, LCN16_00890 and LCN16_03500; *menF*, LCN16_03345; and *pchB*, LCN16_03570), and iron uptake and transport systems (*fhuDBC*, *efeBOU*, and *feoABC*), including 24 genes in the iron complex transporter system (Additional file 4: Table S3). The main secretion systems (TSS) in *Serratia* sp. LCN16 are TSS1 (*tolC*, LCN16_04212; *hasDEF*, LCN16_01553-5) and TSS2, universal Sec-dependent (secretion) and Tat-independent (two-arginine translocation) proteins export systems, from which bacterial Type II toxins (membrane damaging) such as hemolysins (*hpmA*, LCN16_04426; *hlyIII*, LCN16_04002; *tlh*, LCN16_04200), phospholipase C (*plcC*, LCN16_04159), and serralysins (LCN16_00223; LCN16_02109; LCN16_03865) are secreted. In addition to the antimicrobial/antibiotic metabolism genes found in genomic islands described above, the *Serratia* sp. LCN16 genome encodes genes that could be involved in hydrogen cyanide synthesis (*hcnABC*, LCN16_01840-2), and complete gene sets for drug resistance such as beta-lactam (*ampC*) or macrolide (MacAB-TolC transporter), and multiple antibiotic resistance proteins (*marC*, LCN16_02155) (Additional file 4: Table S3).

**Table 1:** List of predicted genes involved in bacterial endophytic behavior [38] in *Serratia* sp. LCN16 genome. *Burkholderia phytofirmans* PsJN (CP001052-54) was used as reference genome for orthologous search in *Serratia* sp. LCN16, *Serratia proteamaculans* 568 (Spro568, NC_009832) and *S. liquefaciens* ATCC 27592 (CP006252). The description presented is based on KEGG annotation [69]

Gene Function	Description	Gene Identification			Orthologous genes		
		Number	Copies	Name	PsJN	Spro568	ATCC 27592
Transporter	Arabinose operon regulatory	LCN16_02277	1	*araC*	Bphyt_0033	Spro_1385	M495_06380
	Lysine exporter protein	LCN16_04024	1		Bphyt_0034	-	M495_06375
	High-affinity branched-chain amino acid transport	LCN16_00248	1	*livF*	Bphyt_3906	Spro_0232	M495_01020
	High-affinity branched-chain amino acid transport	LCN16_00245	1	*livH*	Bphyt_3908	Spro_3202	M495_01005
	NAD(P) transhydrogenase subunit beta	LCN16_02610	1	*pntB*	Bphyt_4261	Spro_2584	M495_12845
	ABC transporter related	LCN16_02535	1	*malk_1*	Bphyt_4584	Spro_4470	M495_22510
	Metabolite:H+ symporter (MHS) family	LCN16_03836	1	*citA*	Bphyt_5520	Spro_3179	M495_10135
	Extracellular solute-binding protein	LCN16_01236	1	*modA*	Bphyt_5521	Spro_3180	-
	Gluconate 2-dehydrogenase	LCN16_02150	1		Bphyt_4638	Spro_2138	M495_10390
	Gluconate 2-dehydrogenase	LCN16_02151	1		Bphyt_4639	Spro_2137	M495_10385
	Gluconate 2-dehydrogenase	LCN16_02152	1		Bphyt_4640	Spro_2136	M495_10380
Secretion and delivery system	TypeVI secretion protein	-	0	-	Bphyt_4913	Spro_3003	-
	TypeVI secretion protein	-	0	-	Bphyt_4914	Spro_3004	-
	TypeVI secretion protein	-	0	-	Bphyt_4919	Spro_3013	M495_03685
	RND family efflux transporter MFP subunit	LCN16_01039	1	*acrA*	Bphyt_6992	Spro_1127	M495_04880
Plant polymer degradation/modification	Alpha/beta hydrolase family protein	LCN16_01434	1		Bphyt_6134	Spro_0990	M495_12205
	Alpha/alpha-trehalase	-	0		Bphyt_5350	-	-
	Cupin	LCN16_02559	2		Bphyt_2288	-	-
	Peptidase M48 Ste24p	LCN16_04051	1	*loiP*	Bphyt_3335	Spro_3955	M495_20655
Transcriptional regulator	HTH-type transcriptional regulator LrpC	LCN16_01418	1	*lrpC*	Bphyt_0434	Spro_1462	M495_06820
	Regulator protein FrmR	LCN16_01244	1	*frmR*	Bphyt_0109	-	-
	AraC family transcriptional regulator	LCN16_02277	1	*araC*	Bphyt_2287	Spro_2540	M495_12625
	Transcriptional regulatory protein	LCN16_03523	1	*ompR*	Bphyt_4604	Spro_4621	M495_23305
	Transcriptional regulatory, DeoR family	LCN16_01600	1	*deoR*	Bphyt_4951	Spro_2259	M495_11240
	Transcriptional regulatory, LysR family	LCN16_02297	1	*ampR*	Bphyt_5523	Spro_3181	M495_17720
	LrgB family operon	-	0		Bphyt_5345	Spro_1569	M495_07365
	Flavoprotein WrbA	LCN16_01736	1	*wrbA*	Bphyt_6351	Spro_1813	M495_08400
Detoxification	Glutathione S-transferase	LCN16_01390	7	*gst*	Bphyt_1366	Spro_3320	M495_17060
	Short-chain dehydrogenase	LCN16_02779	1		Bphyt_1098	Spro_1971	M495_09250
	S-(hydroxymethyl)-gluthathione dehydrogenase	LCN16_01515	1	*frmA*	Bphyt_5114	Spro_1557	M495_07305
	2-hydropantoate 2-reductase	LCN16_00995	1	*panE*	Bphyt_5159	Spro_3174	-
Redox potential maintenance	Acetoacetyl-coa reductase	LCN16_01349	1	*phbB*	Bphyt_5655	Spro_3465	M495_17855
	Acetaldehyde dehydrogenase	LCN16_02742	1	*adhE*	Bphyt_1467	Spro_3026	M495_05210
	Carbonate dehydratase	LCN16_00514	1	*cynT*	Bphyt_2146	Spro_1534	M495_07235
	Aldehyde dehydrogenase	LCN16_02563	3	*gabD*	Bphyt_4023	Spro_4305	M495_21680
	Malate/L-lactate dehydrogenase	LCN16_02031	1	*ybiC*	Bphyt_5456	Spro_2010	M495_09840
	3-hydroxyisobutyrate dehydrogenase	LCN16_01348	1	*garR*	Bphyt_5931	Spro_1492	M495_07025
Others	Amino-acid metabolite efflux pump	LCN16_01419	1	*eamA*	Bphyt_0435	Spro_1463	M495_06825
	2-isopropylmalate synthase	LCN16_00673	2	*leuA*	Bphyt_0573	Spro_1875	M495_00910
	Diaminopimelate decarboxylase	LCN16_03946	1	*lysA*	Bphyt_7089	Spro_3836	M495_20025

### *Serratia* sp. LCN16 tolerance to oxidative stress

*Serratia* sp. LCN16 has been reported as highly tolerant to oxidative stress, exerting a beneficial effect towards *B. xylophilus* under stressful conditions in vitro [22]. *Serratia* sp. LCN16 encodes many antioxidant enzymes in its genome (Table 2), including 7 GSTs, 3 SODs, 2 KATs (HPII, *katA*, LCN16_03339; HPI, *katG*, LCN16_03210), 1 AHP (alkyl hydroperoxide), 3 GPXs, 3 GRXs (glutaredoxin), 2 TRXs and 2 TPXs. To examine the potential roles of some of these proteins in the neutralization of oxidant stressors (e.g., H_2_O_2_), two genes were selected for complete gene knockout, namely the H_2_O_2_ transcriptional factor *oxyR* (LCN16_04688), and the enzyme catalase *katA* (LCN16_03339).

**Table 2:** List of predicted genes involved in oxidative stress of *Serratia* sp. LCN16 genome. Genes descriptions based on KEGG [69]

KEGG	EC	Description	Predicted gene	
K00799	2.5.1.18	Glutathione S-transferase	*gst*	LCN16_01390
				LCN16_01491
				LCN16_01648
				LCN16_02242
				LCN16_03108
				LCN16_03382
				LCN16_04377
K03782	1.11.1.21	Catalase-peroxidase	*katG*	LCN16_03210
K03781	1.11.1.6	Catalase	*katA*	LCN16_03339
K04565	1.15.1.1	Superoxide dismutase Cu-Zn	*sod1*	LCN16_02232
K04564	1.15.1.1	Superoxide dismutase Fe-Mn	*sod2*	LCN16_00084
K04564	1.15.1.1	Superoxide dismutase Fe-Mn	*sod2*	LCN16_02218
K00432	1.11.1.9	Glutathione peroxidase	*gpx*	LCN16_02167
			*gpx*	LCN16_02187
			*gpx*	LCN16_04689
K00384	1.8.1.9	Thioredoxin	*trxB*	LCN16_01650
			*trx*	LCN16_00969
K00383	1.8.1.7	Glutathione reductase	*gorA*	LCN16_04647
K03674	1.20.4.1	Glutaredoxin	*grxA*	LCN16_01610
K07390	1.20.4.2		*grxD*	LCN16_02220
K03675	1.20.4.3		*grxB*	LCN16_02844
K03386	1.11.1.15	Alkyl hydroperoxide	*ahpD*	LCN16_03826
K04761	-	Hydrogen-peroxide transcriptional regulator	*oxyR*	LCN16_04688
K11065	1.11.1.-	Thiol peroxidase	*tpx_1*	LCN16_02660
			*tpx_2*	LCN16_03583
-	-	Organic hydroperoxide resistance transcriptional regulator	*ohrR*	LCN16_00141
-	-	Organic hydroperoxide resistance protein	*ohrB*	LCN16_00142
K13892	-	Glutathione ABC transporter	*gsiA*	LCN16_01509
K13889	-		*gsiB*	LCN16_01510
K13890	-		*gsiC*	LCN16_01511
K13891	-		*gsiD*	LCN16_01512
K01919	6.3.2.2	Glutamate-cysteine ligase	*gshA*	LCN16_00781
K18592	2.3.2.2	Gamma-glutamyltranspeptidase	*ggt_1*	LCN16_00968
			*ggt_2*	LCN16_02503
K00430	1.8.-.-	Thiol-disulfide oxidoreductase	*ykuV*	LCN16_04070

The nucleotide sequence of the LCN16 *oxyR* gene (LCN16_04688) is 918 bp long and is located between *fabR* (LCN16_4686), a predicted HTH-type transcriptional repressor protein, and LCN16_04691, a predicted glutathione peroxidase-like protein. The *oxyR* sequence encodes a 34 kDa unstable protein (305 a.a.) with a predicted LysR-type HTH domain (PROSITE: PS50931). The OxyR protein was 100 % identical to the orthologous sequences from *S. proteamaculans* 568 and *S. grimessii* CR62_05005, 99 % identical to the *S. liquefaciens* ATCC 27592, and shares 88 % identity with the *E. coli* K-12 protein. The *Serratia* sp. LCN16 catalase (*katA*) gene (LCN16_03339) is 1437 bp long, and is located between LCN16_03338, a predicted *yfaZ* precursor, and a cluster of genes *menECBHDF* (LCN16_03339-03345), presumably involved in the menaquinone (vitamin K12) biosynthesis. This gene encodes a 54 kDa stable protein (478 a.a.) with a catalase_3 domain (PROSITE: PS51402), and shares 99 % identity with *S. grimessii* CR62_05005, 98 % with *S. proteamaculans* 568 and *S. liquefaciens* ATCC 27592, and only 42 % identity with *E. coli* K. 12 [37].

**Fig. 3:**
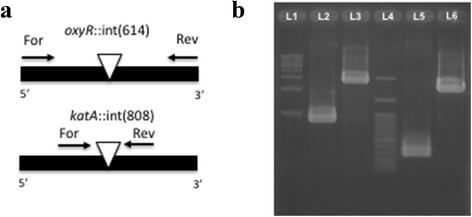
Colony PCR results indicating that group II intron-based vectors successfully targeted the *Serratia* sp. LCN16 *oxyR* and *kat* genes. **a** Introns were inserted in the position 614|615 of *oxyR* gene, *oxyR*::int(614); and in the position 808|809 of *katA* gene, *katA*::int(808). For both genes, the arrows indicate the position of forward and reverse primers designed to infer intron insertion. **b** L1 and L4 indicate 100-bp and 1-kb molecular markers, respectively. L2 and L5 correspond to the *oxyR* (846 bp) and *katA* (292 bp) fragments. L3 and L6 correspond to mobile group II intron integrated in *oxyR* (about 3Kb) and *katA* (about 2-3Kb), respectively

Using TargeTron® (Sigma-Aldrich, MO, St. Louis), a mobile group II intron was modified (retargeted) to be specifically inserted into *oxyR* and *katA* genes in *Serratia* sp. LCN16. The insertion of the retargeted introns (with an approximately size of 2Kb) in the predicted positions, *Serratia* sp. LCN16 *oxyR*::int(614) and *Serratia* sp. LCN16 *katA*::int(808), were confirmed by PCR (Fig. 3a and b). As foreseen in *S. proteamaculans* 568 genome through OperonDB and OperonDetection tools [38, 39], we also predict that, in *Serratia* sp. LCN16, both genes are independently transcribed (not included in an operon-like structure), which may indicate that the *Serratia* sp. LCN16 mutants’ phenotype are only due to these mutated genes.

**Fig. 4:**
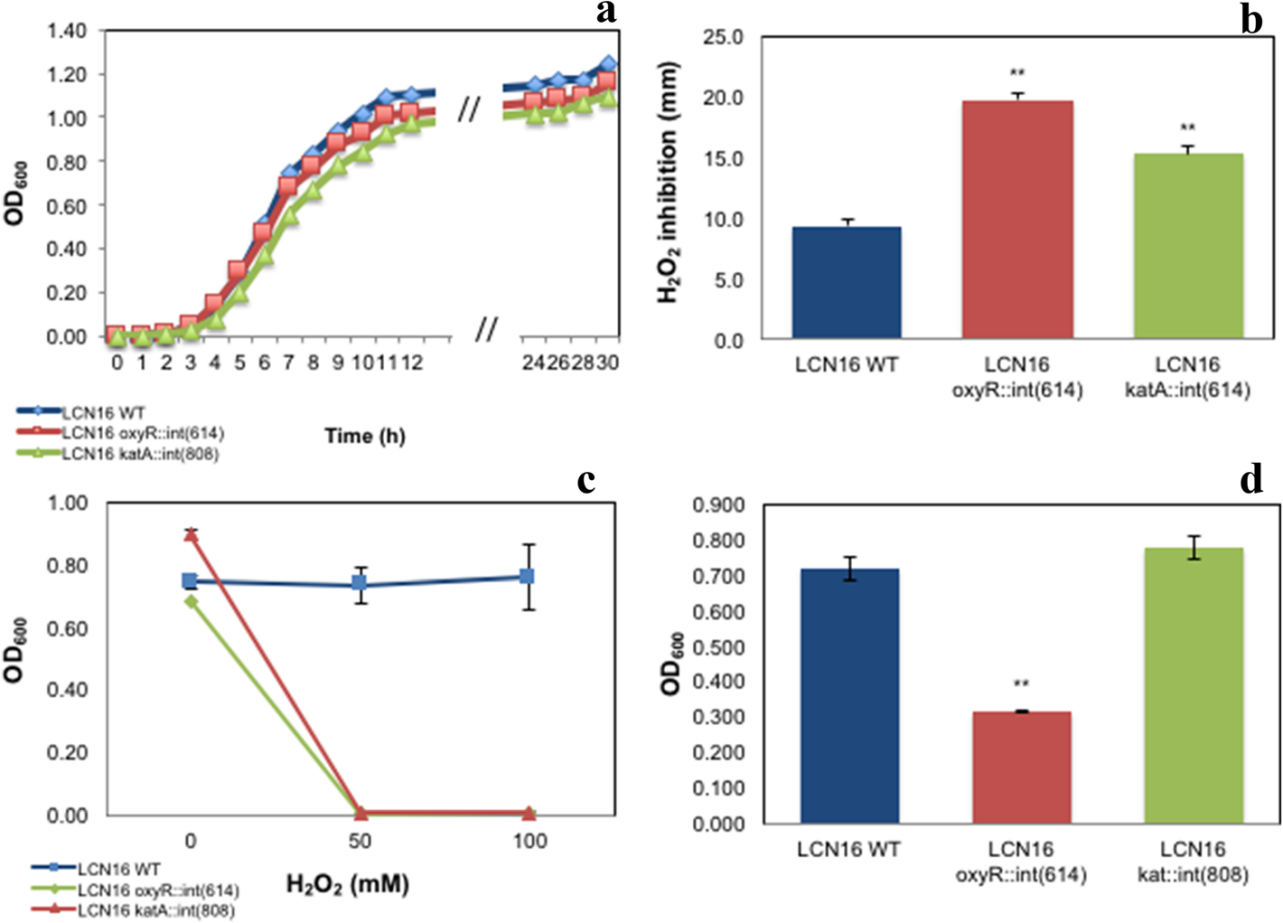
Characterization of H_2_O_2_-sensitive *Serratia* sp. LCN16 (*oxyR*::int(614) and *katA*::int(808)) and resistant *Serratia* sp. LCN16 wild-type (WT): growth curves (**a**); H_2_O_2_ inhibition (mm) (**b**); growth in 24 h exposure to H_2_O_2_ (**c**); and biofilm production (**d**). H_2_O_2_ inhibition was determined by measuring the diameter of the halo surrounding the H_2_O_2_ (30 %, v/v) spot-inoculation. Biofilm production was determined as described in [56]. Error bars indicate standard deviation. Asterisk (*, **) on the top of the columns denotes statistical differences at 95–99 % confidence level by Students *T*-test (EXCEL version 15.14), when compared with wild-type *Serratia* sp. LCN16

The growth of *Serratia* sp. LCN16 WT and mutants is shown in Fig. 4a. Slightly changes were observed in wild-type and mutants’ generation times. The generation time of *Serratia* sp. LCN16 WT was 1.2 h, and the generation times of *Serratia* sp. LCN16 *katA*::int(808) and *oxyR*::int(614) were, respectively, 1.1 h and 1.4 h. The tolerance to H_2_O_2_ was considerably affected in the *oxyR* and *katA* mutants (Fig. 4b, c). The H_2_O_2_ (30 %, w/v; 9.79 M) inhibition was higher in *Serratia* sp. LCN16 *oxyR*::int(614) than in *Serratia* sp. LCN16 *katA*::int(808) (Fig. 4b). Both mutants were statistically different (*P* < 0.01) than the wild-type. At 50 mM and 100 mM H_2_O_2_, both mutants were completely inhibited while *Serratia* sp. LCN16 WT grew easily (Fig. 4c). Consequently, both failed to protect *B. xylophilus* Ka4 after 24 h exposure to 50 mM H_2_O_2_ (Table 3). No statistical differences were seen between *B. xylophilus* Ka4 and *B. xylophilus* Ka4_LCN16 *oxyR*::int(614)/LCN16 *katA*::int(808) (*P* > 0.05), with mortality ranging between 94 and 99 %. Only *B. xylophilus* Ka4_LCN16 WT could reduce significantly (*P* < 0.01) *B. xylophilus* Ka4 mortality to 0.2 %.

**Table 3:** Mortality of *Bursaphelenchus xylophilus* Ka4, alone or in association with *Serratia* sp. LCN16 WT and mutants (*Serratia* sp. LCN16 *oxyR*::int(614) and *Serratia* sp. *kat*::int(808)), in H_2_O_2_ conditions (0 and 50 mM). Statistical differences between treatment Ka4 and the other treatments (Ka4_LCN16 WT; Ka4_LCN16 *oxyR*::int(614); and Ka4_LCN16 *kat*::int(808)) were calculated using Students *T*-test (EXCEL version 15.14). Asterisk (**) denotes statistical differences at 99 % confidence level

Treatment	H_2_O_2_ (mM)	Mortality		*P*-value
		Mean	S.D.	
Ka4	0 mM	0.01	0.00	
Ka4	50 mM	0.99	0.01	
Ka4_LCN16 WT		0.02**	0.00	0.00
Ka4_LCN16 *oxyR*::int(614)		0.94	0.03	0.06
Ka4_LCN16 *kat*::int(808)		0.96	0.07	0.49

**Fig. 5:**
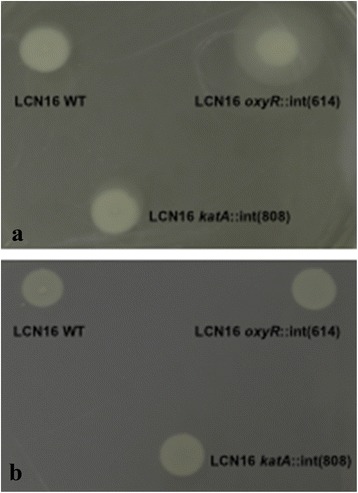
Swimming (**a**) and Swarming (**b**) behaviors of H_2_O_2_-sensitive mutants *Serratia* sp. LCN16 *oxyR*::int(614) and *katA*::int(808) and resistant *Serratia* sp. LCN16 WT. Only *Serratia* sp. LCN16 *oxyR*::int(614) change the swimming behavior as it grown further in the semisolid medium. No effects were seen in swarming behaviors of mutants and wild-type (WT)

Biofilm production (Fig. 4d) was only compromised in *Serratia* sp. LCN16 *oxyR*::int(614), with a significant reduction (*P* < 0.01) in comparison with *Serratia* sp. LCN16 WT. No significant differences (*P* > 0.05) were seen between *Serratia* sp. LCN16 WT and *Serratia* sp. LCN16 *katA*::int(808). In terms of swimming and swarming abilities (Fig. 5), only *Serratia* sp. *oxyR*::int(614) swimming trait was improved.

**Fig. 6:**
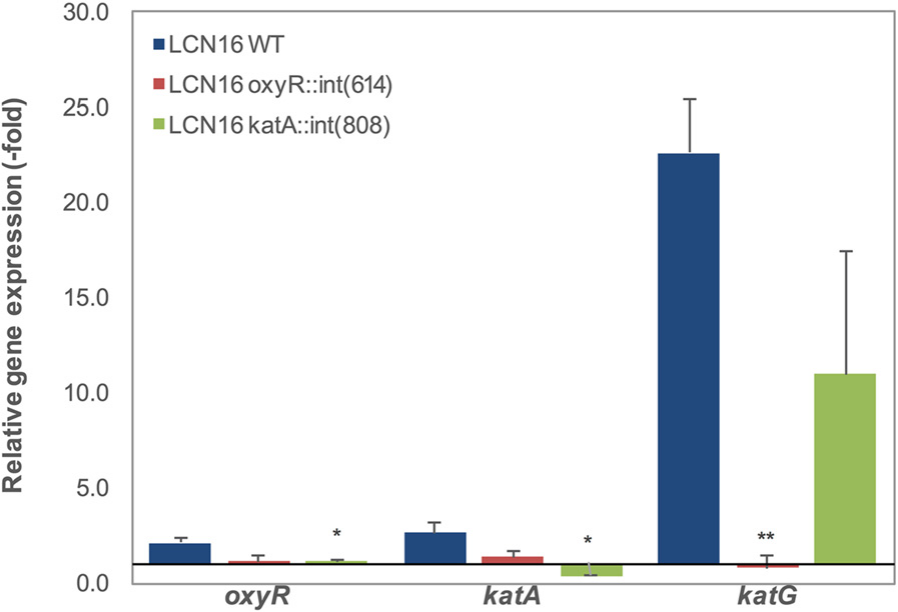
Relative gene expression of *oxyR*, *katA* and *katG* genes in wild-type *Serratia* sp. LCN16 (WT) and *Serratia* sp. LCN16 mutants (*oxyR*::int(614) and *katA*::int(808)), after H_2_O_2_-shock induction. The x-axis was positioned for 1.0, which indicates that the level of gene expression between stress and non-stress conditions is similar. Values were normalized using reference gene *gyrA*, and analyzed with ΔΔC_T_ method. Error bars indicate standard deviation. Asterisk (*, **) on the top of the columns denotes statistical differences at 95–99 % confidence level by Students *T*-test (EXCEL version 15.14), when compared with wild-type *Serratia* sp. LCN16

Relative gene expression of *oxyR*, *katA*, *katG* was analyzed in mid-log phase for the *Serratia* sp. LCN16 WT and mutants (Fig. 6). In terms of the relative expression of *oxyR* (stress versus non-stress conditions), only *Serratia* sp. LCN16 WT showed a 2.2-fold induction, while for LCN16 *oxyR*::(614) and LCN16 *katA*::(808) oxyR the expression levels remained unchanged. Statistical differences (*P* < 0.05) were only detected comparing relative expression of *oxyR* of WT and LCN16 *katA*::(808). For the relative expression of *katA*, only *Serratia* sp. LCN16 WT showed a 2.7-fold induction*.* The relative expression of *katA* in LCN16 *oxyR*::(614) was almost similar between stress and non-stress conditions with a slight induction of 0.4-fold. For the LCN16 *katA*::(808) mutant, *katA* expression was almost null indicating successful mutation of this gene. Statistical differences (*P* < 0.05) were only seen between WT and LCN16 *katA*::(808). The relative gene expression of *katG* was considerably high in LCN16 WT and LCN16 *katA*::(808), respectively 22.6-fold and 11.0-fold. Since *katG* is under regulation of OxyR, its expression is supposed to be comprised in LCN16 *oxyR*::(614) mutant. Thus, the relative expression of *katG* in LCN16 *oxyR*::(614) was equal between stress and non-stress conditions and statistically different (*P* < 0.01) from WT.

## Discussion

Forest trees harbor population densities of endophytic bacteria ranging between 10^1^ and 10^6^ CFU (colony forming unit) per gram of sample, most of which are host-specific [40, 41]. Genomes of plant-associated bacteria reflect a wide spectrum of life style adaptations [36]. Therefore, we cannot dismiss the potential of bacteria when we are trying to understand a particular ecosystem. In the present study, we characterized the biology of *Serratia* sp. LCN16 analyzing, in detail, its genome content, and investigated the role OxyR and KatA in the extreme oxidative stress resistance of this PWN-associated bacteria.

Members of the genus *Serratia* are ecological generalists found inhabiting water, soil, plants, animals and humans [24]. Belonging to the phytosphere *Serratia* group [24], also known as *Serratia liquefaciens* complex [29], *Serratia* sp. LCN16 is phylogenetically closest to the poplar endophyte *S. proteamaculans* 568 [36] and *S. liquefaciens* strain ATCC 27592 [42]. However, this taxonomical identification may be incomplete due to the lack of complete genome sequence for other *Serratia* such as *S. grimessi* strain A2 [43], which is also included in the *S. liquefaciens* complex [30]. *Serratia* sp. LCN16 shares many features with endophytic bacteria, such as plant polymer degrading enzymes, iron uptake/transport, siderophore and phytohormone (i.e., IAA) synthesis, and aromatic compound degradation [33], some of which are supported by the previous phenotypic characterization [16]. Wilted pine trees are a rich source of fungi, which in the late-stages of PWD are consumed by PWN [2]. *Serratia* sp. LCN16, isolated from the cuticle of fungi-cultivated *B. xylophilus* [44], harbors genes involved in the production of antifungal agents (i.e., pyrrolnitrin) and chitinases, which may explain its survival and persistence in *B. xylophilus* lab-culture. Genes encoding other antimicrobial compounds were found in *Serratia* sp. LCN16, which can give a fitness advantage to this bacterium in a more complex environment such as host pine trees [45]. Few MGIs were found in *Serratia* sp. LCN16 suggesting a more stable genome probably adapted to a less broad environment as described elsewhere [34, 46, 47].

Endophytes have highly elaborate detoxification mechanisms to counter-attack host ROS [35, 48]. Vicente et al. [22] showed that *Serratia* sp. LCN16 is highly H_2_O_2_-tolerant bacterium. A total of 16 antioxidant enzymes were found in the *Serratia* sp. LCN16 genome, which is within the range described in other endophytes (from 8 in *Azospirillum* sp. B510 to 21 in *Burkholderia phytofirmans* PsJN) [35]. *Serratia* sp. LCN16 is a copious siderophore producer [16], rich in iron uptake and transport systems (i.e., multiple copies for iron III complex transporter) and, recently, Li et al. [49] reported the importance of siderophore synthesis and iron uptake systems in the resistance against oxidative stress of insect-gut *Elizabethkingia anophelis* NUHP1. The relation between oxidative stress and siderophore synthesis has also been explored in the fungus *Alternaria alternata* [50] and in *Aspergillus nidulans* [51].

Mobile group II introns are retroelements, which through retrohoming mechanism, can be inserted into a DNA target site in a site-specific manner via the activity of an associated intron-encoded enzyme protein [52]. Wang et al. [53] could efficiently disrupt the acid production pathways in *Clostridium beijinrinckii* by the insertion of group II intron specifically retargeted to *pta* (encoding phosphotransacetylase) and *buk* (encoding butyrate kinase). Using this gene knockout system, we obtained two mutants, *Serratia* sp. LCN16 *oxyR*::int(614) and *Serratia* sp. LCN16 *katA*::int(808), disrupting respectively, *oxyR* and *katA* genes. Both genes have been investigated for their direct involvement in oxidative stress in several organisms [54–59]. The OxyR is a global regulator of peroxide stress response that maintains intracellular H_2_O_2_ homeostasis, and it is known to regulate several genes involved in H_2_O_2_ detoxification (i.e., *katG*, *grxA*, *ahpCF* and *trxC*) [54, 55]. The *Serratia* sp. LCN16 *oxyR*::int(614) was severely impaired in its ability to survive in high H_2_O_2_ conditions in vitro, highly affected in biofilm production (production decreased) and swimming abilities. Our results are corroborated by Shank et al. [56], which previously observed that *Serratia marcescens* OxyR mediated oxidative stress response, biofilm formation and surface attachment. The high sequence similarity between *Serratia* sp. LCN16 OxyR and *E. coli* K12 (88 %) suggests that *Serratia* sp. LCN16 utilizes a similar mechanism of regulation as *E. coli*. Under high H_2_O_2_ stress conditions, OxyR (reduced/inactive form) is oxidized triggering conformational changes (OxyR oxidized/active form) leading to the regulation of several proteins including the expression of KatG [55]. This effect was clearly seen in our results. The expression of *katG* was significantly down regulated in *Serratia* sp. LCN16 *oxyR*::int(614) (Fig. 6), suggesting its OxyR-dependent regulation. The importance of KatG has been observed in several studies. Jamet et al. [57] observed the role of *katG* as a mediator of bacteria-plant interaction for *Sinorhizobium meliloti*, reporting its constitutive expression and considering *katG* to be a housekeeping gene. *Mycobacterium tuberculosis*, an opportunistic bacterial pathogen, also relies on KatG for its resistance to high oxidative stress environments [58]. *Serratia* sp. LCN16 encodes also other catalase, *katA* (mono-functional), which is homologous with *E. coli katE. Serratia* sp. LCN16 *katA*::int(808) was affected in H_2_O_2_ resistance but not in biofilm production nor swimming and swarming traits. The expression of the *katA* in *Serratia* sp. LCN16 WT was slightly induced when compared with *katG*, indicating that its expression in exponential phase is reduced. In *oxyR* mutant, *katA* expression was relatively closest to the expression level showed in wildtype, suggesting to be regulated in a OxyR-independent manner. In *E. coli*, *katE* is transcriptionally regulated in the stationary phase by RpoS [55]. As for KatG, KatA was also found important in bacteria-plant interaction [60].

## Conclusions

The present study revealed the potential of PWN-associated bacteria *Serratia* sp. LCN16 to live in a plant-environment, and also that its high tolerance to oxidative stress is OxyR- and KatA-dependent. In the PWD context, we showed that *Serratia* sp. LCN16 *oxyR*::int(614) and *Serratia* sp. LCN16 *katA*::int(808) failed to protect PWN against H_2_O_2_ oxidative conditions. As previously hypothesized [22], PWN-associated bacteria may opportunistically assist the nematode in the disease by amelioration of oxidative burst of pine defenses. Through this study, we have set the proper conditions to explore bacteria-nematode association *in planta* environment.

## Methods

### Bacterial strain

*Serratia* sp. LCN16 was isolated from the cuticle of lab-culture *Bursaphelenchus xylophilus* isolate Bx153-3A (Setubal Peninsula, Portugal) [16]. This bacterium belongs to the bacterial culture collection of NemaLab/ICAAM (Évora, Portugal), and is maintained in 30 % (w/v) glycerol stocks at −80 °C. For all experiments, *Serratia* sp. LCN16 was recovered from long-term stock and grown in LB (Luria-Bertani) for 1 day at 28 °C.

### Genome sequencing, annotation and analysis

A single colony of *Serratia* sp. LCN16 was used to inoculate 10 ml of LB and incubated overnight at 28 °C with shaking. Genomic DNA was extracted from the overnight culture using the QIAGEN Genomic DNA Purification kit (Qiagen), following the manufacturer’s instructions. This DNA was sequenced on the Roche Titanium 454 platform at the Centre for Genomic Research, University of Liverpool, with large-insert 3 kb paired end libraries. This gave HYMXIQB02 (ENA accession ERS980300) and HYMXIQB03.sff (ENA accession ERS980301) with in total 607,360 sequences, mean length 497.0 bp, median length 465 bp. Initial assemblies were performed with Roche “Newbler” gsAssembler [61], and MIRA v4.0.2 [62]. This data was supplemented with 169,073 paired reads of mean length 132.6 bp from an Illumina MiSeq commissioning test run at the James Hutton Institute (ENA accession ERS980302), as one of 11 barcoded samples. Again, multiple assemblies were evaluated. The final assembly selected was a hybrid 454 and MiSeq assembly using the MIRA v4.0.2, which resolved some of the homopolymer errors detected in the initial 454 assemblies. The genome sequence of *Serratia* sp. LCN16 is available in the European Nucleotide Archive (ENA) under the accession ERP013273.

Genome annotation was performed using PROKKA [63], and manually reviewed in ARTEMIS [64] (ENA accession ERS1015427). The circular genome image was plotted in DNA PLOTTER [65]. Protein annotation (InterPro and Gene Ontology) was further supported by BLAST2GO [66] and KAAS (KEGG Automatic Annotation Server) [67]. Genomic islands were annotated using online-tool Island Viewer 3.0 [31]. Genome to genome alignments of *Serratia* sp. LCN16, *Serratia proteamaculans* 568 (CP000826.1) and *Serratia liquefaciens* ATCC 27592 (CP006252.1) were conducted using MAUVE software [68]. The 16S rRNA and four housekeeping genes (*atpD*, *dnaJ*, *gyrB* and *rpoB*) were used to infer the phylogenetic relationship between *Serratia* sp. LCN16 and the following *Serratia*-type strains: *S. liquefaciens* ATCC 27592, *S. marcescens subsp. marcescens* Db11 (NZ_HG326223), *S. marcescens* WW4 (CP003959.1), *S. plymuthica* 4RX13 (CP006250.1), *S. proteamaculans* 568, and *S. symbiotica* “Cinara cedri” (CP0022951.1). *Bacillus subtilis* XF1 (CP004091.1) was used as out-group. Housekeeping sequence genes were concatenated using Seaview 4.0 [69]. All phylogenetic analyses were conducted in MEGA6 [70]. Phylogenetic robustness was inferred by bootstrap analysis using 1,000 iterations.

### Functional analysis of oxidative stress resistance of *Serratia sp.* LCN16

#### Strains, plasmids, media and growth

All bacteria and plasmids used in this study are listed in Table 4. All bacteria were grown in LB at 28 °C and 200 rpm. The antibiotics used in this study were gentamicin (10 μg/ml and 30 μg/ml), chloramphenicol (50 μg/ml), kanamycin (50 μg/ml), and ampicillin (100 μg/ml).

**Table 4:** List of strains, plasmids and primers used in the present study

Strain, plasmid and primers	Genotype or phenotype
*Serratia* sp.	
LCN16 WT	WT resistant to ampicillin and erythromycin.
LCN16 *oxyR*::int(614)	Knockout mutant of *oxyR* with group II intron inserted in the position 614; Resistant to gentamicin and kanamycin.
LCN16 *katA*::int(808)	Knockout mutant of *katA* with group II intron inserted in the position 808*;* Resistant to gentamicin and kanamycin.
*Escherichia coli*	
DH5α	Competent cells (Sigma-Albrich)
Plasmids	
pAR1219	TargeTron vector with chloramphenicol resistant
pAR1219-ΩGm	Constructed vector with gentamicin resistant gene *aacC*
pACD4K-C	TargeTron vector; kanamicin RAM marker (for chromosomal insertion) and chloramphenicol resistance (plasmid propagation)
pACD4K-C_oxyR	TargeTron vector with intron RNA retarget for *oxyR* gene
pACD4K-C_kat	TargeTron vector with intron RNA retarget for *ka*t gene
pBK-miniTn7-ΩGm	Tn7 plasmid constructed with gentamicin resistant gene *aacC*
PCR Primers (5′-3′)	
aacC1_IfFor	CATACTCTTCCTTTTTCAATATTATTG
aacC1_IfRev	TAACTGTCAGACCAAGTTTACTC
oxyR_614|615 s-IBS	AAAAAAGCTTATAATTATCCTTAGGAAGCTGGTCAGTGCGCCCAGATAGGGTG
oxyR_614|615 s-EBS1d	CAGATTGTACAAATGTGGTGATAACAGATAAGTCTGGTCACTTAACTTACCTTTCTTTGT
oxyR_614|615 s-EBS2	TGAACGCAAGTTTCTAATTTCGATTCTTCCTCGATAGAGGAAAGTGTCT
oxyR_KO_chkFor	CGTGGTCTGGAGGGAAACAA
oxyR_KO_chkRev	CATAACGACTGCGCAATGGG
katA_808|809a -IBS	AAAAAAGCTTATAATTATCCTTATAATCCGGATTTGTGCGCCCAGATAGGGTG
katA_808|809a EBS1d	CAGATTGTACAAATGTGGTGATAACAGATAAGTCGGATTTGCTAACTTACCTTTCTTTGT
katA_808|809a -EBS2	TGAACGCAAGTTTCTAATTTCGGTTGATTATCGATAGAGGAAAGTGTCT
katA_KO_chkFor	GGTGAAGTTCCATTTCCGCTGC
katA_KO_chkRev	GGGTTCACCGCTACCTGTTCAAC
RT-qPCR primers (5′-3′)	
gyrA_For	TTATCTCCCTGATTGTGCCA
gyrA_Rev	CATTACGCTCGCTCACCTTA
oxyR_For	TTTAGAGTACCTGGTCGCCTTG
oxyF_Rev	ATCACACCCAGTTCGTCTTCC
katA_For	CCAGATTATGCCTGAACACG
katA_Rev	TGCAGTTCGAAGAAACCAAC
katG_For	AGCGGTAAGCCAAATACACC
katG_Rev	AATCGAAGTCAGGGTCCATC

### Gene knockout of *oxyR* and *kat*

The TargeTron® Gene Knockout System from Sigma-Aldrich (St. Loius, MO) was used to obtain complete gene knockouts of transcription factor OxyR and catalase KatA from *Serratia* sp. LCN16. The mobile group II intron sites for *oxyR* (LCN16_04688) and *katA* (LCN16_03339) were predicted using online TargeTron Design site (Sigma-Aldrich, St. Louis, MO). Intron PCR template was retargeted for both genes using the primers designed in the online tool and listed in Table 4. For *oxyR* gene, the following four primers were used: EBS universal primer, *oxyR_*614|615 s-IBS, *oxyR_*614*|*615 s-EBS1d, and *oxyR_*614|615 s-EBS2. For *katA*, the four primers used were: EBS universal primer, *katA_*808|809a-IBS, *katA_*808|809a-EBS1d, and *katA_*808|809a-EBS2. The amplified 350-bp DNA fragment was double digested with *Hind* III and *BsrG* I and ligated into the linear vector pACD4K-C (pACD4K-C_oxyR and pACD4K-C_katA, Table 4). Both plasmids were cloned in *E. coli* DH5α competent cells, following the manufacture’s instructions (Invitrogen). Since *Serratia* sp. LCN16 WT (wild type) is resistant to ampicillin, the plasmid pAR1219-ΩGm was constructed using pAR1219 linear without marker, previously amplified from the pAR1219 (Sigma-Aldrich, St. Louis, MO) using pAR1219_LFor and pAR1219_LRev, with gentamicin resistance used as an alternative antibiotic marker. Briefly, aaaC1 (gentamicin cassette) was amplified from pBK-miniTn7-ΩGm using aacC1_IfFor and aacC1_IfRev primers (Table 4). The PCR product was ligated into the pAR1219 linear with In-fusion® HD cloning system (Clontech Laboratories). The resultant plasmid was also cloned into *E. coli* DH5α competent cells, following the manufacture’s instructions (Invitrogen). TargeTron plasmids isolated from the correct clones were transformed sequentially into electrocompetent *Serratia* sp. LCN16 through electroporation. For each transformation, 50 μl of electrocompetent *Serratia* sp. LCN16 suspension was mixed with 5 μl of plasmid DNA and then added into a 0.2-cm precooled electroporation cuvette and incubated 2 min. Electroporation was carried out using the following conditions: 2,500 V of voltage, 25 μF of captaincy and 200 Ω of resistance. Afterwards, cells were incubated in LB for 1 h at 30 °C, and plated on selective medium (pACD4K-C_oxyR and pACD4K-C_kat in LB supplemented with 50 μg/ml of chloramphenicol and 30 μg/ml gentamicin; pAR1219-ΩGm in LB with 30 μg/ml gentamicin). Induction of the gene disruption was conducted in *Serratia* sp. LCN16 WT containing both plasmids (pACD4K-C_oxyR/kat and pAR1219-ΩGm) using 100 mM IPTG during 30 min at 25 °C. Induced cells were then pellet at high speed, suspended in LB, incubated at 25 °C for 1 h, and plated in LB supplemented with 50 μg/ml of kanamycin and 30 μg/ml gentamicin. Kanamycin-resistant colonies were picked for colony PCR to detect intron insertions, using the primers oxyR_KO_chkFor/oxyR_KO_chkRev; and katA_KO_chkFor/katA_KO_chkRev (Table 4). Successful mutants were named *Serratia* sp. LCN16 *oxyR*::int(614) and *Serratia* sp. LCN16 *katA*::int (808).

### Characterization of *Serratia* sp. LCN16

Unless otherwise specified, *Serratia* sp. LCN16 WT, *Serratia* sp. LCN16 *oxyR*::int(614) and *Serratia* sp. LCN16 *katA*::int(808) were grown overnight at 28 °C from a single colony in LB with respective antibiotics, and adjusted to OD_600_ of 0.8.

#### Growth curves

The growth rates of all bacteria were determined in 5 ml of LB incubated at 30 °C with 200 r.p.m (initial OD_600_ 0.05). Triplicate 100 μl aliquots were removed at various time points, and the culture turbidity (OD_600_) was determined using a multi-spectrophotometer 96-well plate reader (Bio-Tek, Synergy H1 microplate reader; Gen5 version 2.05). This experiment was repeated two times in independent days. Generation time (h) was determined in the exponential phase of bacterial growth.

#### Tolerance to H_2_O_2_

*Serratia* sp. LCN16 WT, *Serratia* sp. LCN16 *oxyR*::int(614) and *Serratia* sp. LCN16 *katA*::int(808) were tested for their tolerance to H_2_O_2_, bacteria-only and in association with *B. xylophilus* Ka4. For the bacteria-only test, 100 μl of LB supplemented with H_2_O_2_ (final concentrations of 0, 50 and 100 mM) and 10 μl of overnight bacterial culture were incubated in 96-well plate for 24 h at 30 °C. Bacterial growth was read in a multi-spectrophotometer 96-well plate reader (Bio-Tek, Synergy H1 microplate reader; Gen5 version 2.05). Three independent biological replicates with three technical replicas per experiment were used for each treatment.

To test nematode-bacteria association in H_2_O_2_ conditions (final concentration 50 mM), firstly nematodes were surface-sterilized as described by Takemoto [71], and the concentration was adjusted to 150 nematodes per 50 μl of ddH_2_O. Secondly, nematode-bacteria association was performed by 1 h contact between surface cleaned nematodes and 1 ml of bacteria suspension (prepared as referred above) following the Han et al. [18] procedure. After contact, nematodes were washed and re-suspended in ddH_2_O. A 96-well plate was prepared as follows: each well received 50 μl of H_2_O_2_ suspension and 50 μl of each treatment (nematode-bacteria association and nematode alone). Control treatment of *B. xylophilus* Ka4 with ddH_2_O was also prepared. Three independent biological replicates with two technical replicas per experiment were used for each treatment. Nematode mortality was scored after 24 h. Nematodes were considered dead, if no movements were observed after mechanical stimulation.

#### H_2_O_2_ inhibition, swimming and swarming assays, and biofilm production

H_2_O_2_ inhibition and swimming/swarming assays were tested according to [56] and biofilm production adapted from [72]. For these experiments, three biological replicates with three technical replicas were performed for each bacterium.

H_2_O_2_ inhibition was tested by disk diffusion assays. Overnight bacteria culture was spread on LB medium and a sterile 6-mm paper disk was placed in the center of the plate, to which 10 μl of 30 % H_2_O_2_ (w/v, 9.79 M) was added. Plates were incubated overnight at 28 °C. H_2_O_2_ inhibition was determined by measuring the diameter of the inhibition halo surrounding the H_2_O_2_ disk. Swimming and swarming were tested in LB medium, respectively, with 0.3 %(w/v) and 0.6 %(w/v) agar. Bacteria was inoculated (10 μl) into semi-solid LB and incubated overnight at 28 °C. The ability to swim or swarm was inferred by visualization of colony expansion or shape.

For quantitative evaluation of biofilm production [72], 10 μl overnight bacteria culture (OD_600_ 0.02) was inoculated into 150 μl LB in individual wells of a 96-well plate, and incubated for 48 h at 28 °C. Following incubation, the plate was gently washed with ddH2O, dried at 30 °C for 30 min, and wells filled with 150 μl of 0.1 % crystal violet stain. After 1 h incubation at room temperature, wells were gently rinsed with ddH2O, filled with 180 μl of ETOH (96 %, v/v), and incubated for 20 min at room temperature. The 96-well plate was read in a multi-spectrophotometer 96-well plate reader (Bio-Tek, Synergy H1 microplate reader; Gen5 version 2.05) at 590 nm.

### RNA extraction and Real-time PCR

To understand the gene regulation under oxidative stress conditions, *oxyR*, *katA* and *katG* were selected for gene expression analysis. Predictions about general topology, domain/family and retrieved best matches were made using the online tools (Translate, ScanProsite, ProtParam) at Expasy WWW pages (http://www.expasy.org/).

All bacteria (WT and mutants) were grown in M9 medium supplemented with sucrose (2 % v/v) till mid-log phase (OD_600_ 0.100–0.200, BioTek Synergy H1 multichannel spectrophotometer) and shocked with 50 mM H_2_O_2_ for 5 min. One-ml of each treatment was stabilized in RNA protect (Qiagen) and total RNA was extracted with RNeasy ® Minikit (Qiagen), following the manufacturer’s instructions. The concentration and quality of extracted RNA was measured using NanoVue plus spectrophotometer (GE Healthcare Life Science, USA). Total RNA was, firstly, treated with DNase I (Takara Bio Inc., Japan), adjusted to a final concentration of 500 ng/μl and reverse transcribed using random hexamers primers and PrimeScript RT enzyme from PrimeScript™ RT reagent kit (Perfect Real Time) (Takara Bio Inc., Japan). Quantitative RT-PCR was performed using CFX96™ Real-Time (Bio-Rad), and SYBR Premix Ex TaqTM II (Tli RNAse H Plus) kit (Takara Bio Inc., Japan). The housekeeping *gyrA* gene (LCN16_03331) was used as an internal control gene for the calculation of relative expression levels of each selected gene. Primers were designed using Primer3 software [73] (Table 4) and tested for specificity prior to qPCR. Two independent biological replicates with two technical replicas per experiment were used for each qPCR test. No template (NTC) and RNA controls were prepared for each qPCR run. Thermal cycling conditions were: initial denaturation at 95 °C for 30 s; 39 cycles of denaturation at 95 °C for sec, annealing and extension at 60 °C for 30 s; followed by the melting curve. Relative gene expression of each gene were analyzed using ΔΔC_T_ method [74]. Data were analyzed with C_T_ values in normal and stress conditions and using Eq. 2

2 $$ \Delta \Delta CT=\left(CT,\  target-CT,\  gyrA\right) normal-\left(CT,\  target-CT,\  gyrA\right) stress $$

The fold change of *oxyR*, *katA* and *katG* were normalized to *gyrA* and relative to the expression at normal conditions.

### Statistical analyses

Statistical differences at 95–99 % confidence level between mutants (*Serratia* sp. LCN16 *oxyR*::int(614) and *Serratia* sp. LCN16 *katA*::int(808)) and *Serratia* sp. LCN16 WT were calculated using Student’s *T*-Test in Excel version 15.14.

## Ethics and consent to participate

Not Applicable.

## Availability of Data and Materials

The datasets supporting the conclusions of this article are available in the TreeBase repository (http://purl.org/phylo/treebase/phylows/study/TB2:S19116), in the European Nucleotide Archive (ENA) under the accession ERP013273, and included within the article and its additional files.

## Additional files

Additional file 1: Table S1. Gene ontology of *Serratia* sp. LCN16 according to BLAST2GO [57]. (PDF 20 kb) Additional file 2: Figure S1. Comparison of *Serratia* sp. LCN16 genome and the closest *Serratia* representatives, *S. proteamaculans* 568 (Spro568) and *S. liquefaciens* ATCC 27592. Genome to genome alignment conducted using MAUVE software [64]. S1A figure shows the syntenic regions (locally collinear blocks, LCD) between genomes. In each LCD, the height of the similarity profile indicates the level of conservation in that genome region. White areas indicate specific sequences of the genome. The genome rearrangements are indicated as different colored lines. S1B figure presents the similarities between genomes are indicate as follows: purple indicates conserved regions in all genomes; green indicates *Serratia* sp. LCN16 and ATCC 27592 genomes (highlighted with red arrow); reds indicate *Serratia* sp. LCN16 and Spro568 genomes; and orange, conservation between Spro568 and ATCC 27592. (PDF 3065 kb) Additional file 3: Table S2. List of genes predicted in *Serratia* sp. LCN16 genomic islands (GI). GIs at least one of the two methods SIGI-HMM or IslandPath-DIMOB in IslandViewer server [25]. (PDF 263 kb) Additional file 4: Table S3. List of genes predicted to be involved in a plant-associated life-style of *Serratia* sp. LCN16. (PDF 113 kb)

## Abbreviations

CDS: coding sequences
CFU: colony forming unit
CRISPR: clustered regularly interspaced short palindromic repeats
GI: genomic islands
GO: gene ontology
GPX: glutathione peroxidase
GST: gluthathione S-transferase
H_2_O_2_: hydrogen peroxide
IAA: indole acetic acid
KAT: catalase
MGE: mobile genomic elements
PRX: peroxiredoxin
PWD: pine wilt disease
PWN: pine wood nematode
ROS: reactive oxygen species
SOD: superoxidase dismutase
TPX: thiol peroxidase
TRX: thioredoxin
WT: wild type
