# Supplementary material for: The genome and genetics of a high oxidative stress tolerant Serratia sp. LCN16 isolated from the plant parasitic nematode Bursaphelenchus xylophilus
Source: BMC Genomics. 2016 Apr 23;17:301. doi: 10.1186/s12864-016-2626-1 (PMC4841953; doi:10.1186/s12864-016-2626-1)
Supplement: Additional file 1: Table S1. — Gene ontology of Serratia sp. LCN16 according to BLAST2GO [57]. (PDF 20 kb) [file 12864_2016_2626_MOESM1_ESM.pdf]

Biological Processes - GO-id	GO-term	#Seqs
GO:0008152	metabolic process	1982
GO:0009987	cellular process	1818
GO:0044699	single-organism process	1586
GO:0051179	localization	736
GO:0065007	biological regulation	504
GO:0050896	response to stimulus	212
GO:0071840	cellular component organization or biogenesis	128
GO:0023052	signaling	95
GO:0040011	locomotion	28
GO:0022610	biological adhesion	18
GO:0032502	developmental process	12
GO:0051704	multi-organism process	12
GO:0007610	behavior	1
GO:0000003	reproduction	1
**Molecular Functions - GO-id**	**GO-term**	**#Seqs**
GO:0003824	catalytic activity	1852
GO:0005488	binding	1344
GO:0005215	transporter activity	449
GO:0001071	nucleic acid binding transcription factor activity	293
GO:0060089	molecular transducer activity	80
GO:0005198	structural molecule activity	65
GO:0009055	electron carrier activity	49
GO:0016209	antioxidant activity	19
GO:0000988	protein binding transcription factor activity	12
GO:0098772	molecular function regulator	8
GO:0045182	translation regulator activity	1
GO:0045735	nutrient reservoir activity	1
**Cellular Components - GO-id**	**GO-term**	**#Seqs**
GO:0016020	membrane	892
GO:0005623	cell	535
GO:0032991	macromolecular complex	161
GO:0043226	organelle	88
GO:0005576	extracellular region	5
GO:0031012	extracellular matrix	3
GO:0019012	virion	3
GO:0009295	nucleoid	2
GO:0031974	membrane-enclosed lumen	1
