# Supplementary figures and images for: The genome and genetics of a high oxidative stress tolerant Serratia sp. LCN16 isolated from the plant parasitic nematode Bursaphelenchus xylophilus
Source: BMC Genomics. 2016 Apr 23;17:301. doi: 10.1186/s12864-016-2626-1 (PMC4841953; doi:10.1186/s12864-016-2626-1)

S1A

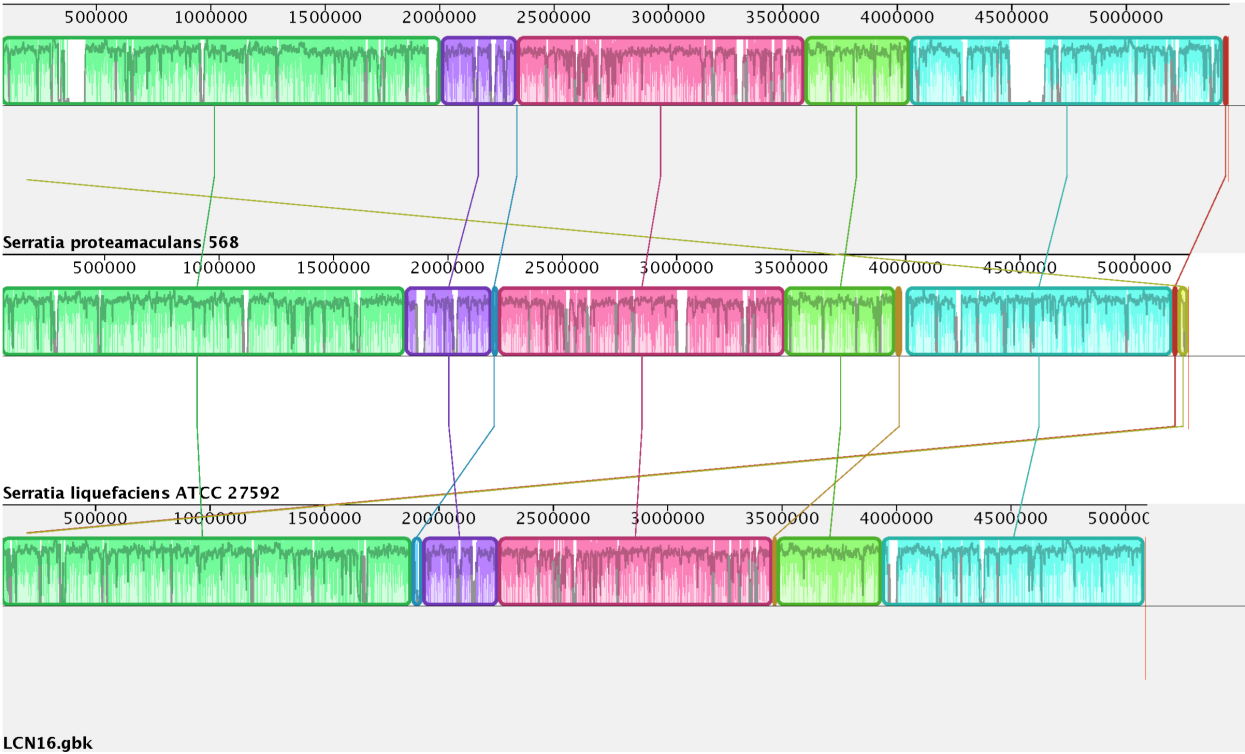

S1B

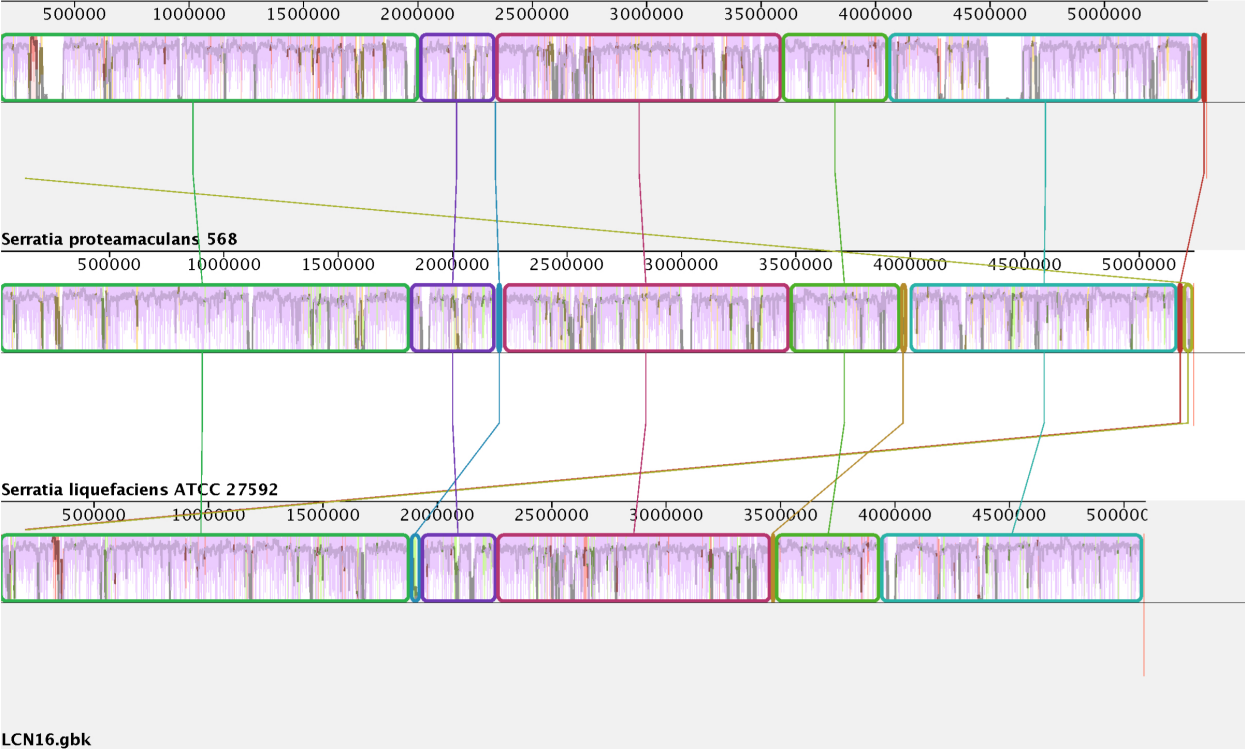

Supplement: Additional file 2: Figure S1. — Comparison of Serratia sp. LCN16 genome and the closest Serratia representatives, S. proteamaculans 568 (Spro568) and S. liquefaciens ATCC 27592. Genome to genome alignment conducted using MAUVE software [64]. S1A figure shows the syntenic regions (locally collinear blocks, LCD) between genomes. In each LCD, the height of the similarity profile indicates the level of conservation in that genome region. White areas indicate specific sequences of the genome. The genome rearrangements are indicated as different colored lines. S1B figure presents the similarities between genomes are indicate as follows: purple indicates conserved regions in all genomes; green indicates Serratia sp. LCN16 and ATCC 27592 genomes (highlighted with red arrow); reds indicate Serratia sp. LCN16 and Spro568 genomes; and orange, conservation between Spro568 and ATCC 27592. (PDF 3065 kb) [file 12864_2016_2626_MOESM2_ESM.pdf]
