# Supplementary material for: The genome and genetics of a high oxidative stress tolerant Serratia sp. LCN16 isolated from the plant parasitic nematode Bursaphelenchus xylophilus
Source: BMC Genomics. 2016 Apr 23;17:301. doi: 10.1186/s12864-016-2626-1 (PMC4841953; doi:10.1186/s12864-016-2626-1)
Supplement: Additional file 3: Table S2. — List of genes predicted in Serratia sp. LCN16 genomic islands (GI). GIs at least one of the two methods SIGI-HMM or IslandPath-DIMOB in IslandViewer server [25]. (PDF 263 kb) [file 12864_2016_2626_MOESM3_ESM.pdf]

Island start	Island end	Length	Method	Gene ID	Locus	Gene start	Gene end	Strand	Product
120058	140469	20411	IslandPath-DIMOB	yycB	LCN16_00106	120058	121257	1	putative transporter YycB
120058	140469	20411	IslandPath-DIMOB		LCN16_00107	121334	122680	1	hypothetical protein
120058	140469	20411	IslandPath-DIMOB	intS	LCN16_00109	123051	124235	1	Putative prophage CPS-53 integrase
120058	140469	20411	IslandPath-DIMOB		LCN16_00110	124232	125062	1	hypothetical protein
120058	140469	20411	IslandPath-DIMOB		LCN16_00111	125183	125479	1	Prophage CP4-57 regulatory protein (AlpA)
120058	140469	20411	IslandPath-DIMOB		LCN16_00112	125826	126002	1	hypothetical protein
120058	140469	20411	IslandPath-DIMOB		LCN16_00113	125995	126348	1	hypothetical protein
120058	140469	20411	IslandPath-DIMOB		LCN16_00114	126393	126674	1	hypothetical protein
120058	140469	20411	IslandPath-DIMOB		LCN16_00115	126671	127021	1	hypothetical protein
120058	140469	20411	IslandPath-DIMOB	traC	LCN16_00116	127031	129712	1	DNA primase TraC
120058	140469	20411	IslandPath-DIMOB		LCN16_00117	130133	130882	1	hypothetical protein
120058	140469	20411	IslandPath-DIMOB		LCN16_00118	130885	131121	1	DNA-binding transcriptional regulator
120058	140469	20411	IslandPath-DIMOB		LCN16_00119	131423	132703	1	Reverse transcriptase (RNA-dependent DNA polymerase)
120058	140469	20411	IslandPath-DIMOB		LCN16_00120	132694	134715	1	Reverse transcriptase (RNA-dependent DNA polymerase)
120058	140469	20411	IslandPath-DIMOB	ydcR_1	LCN16_00121	135248	136702	-1	putative HTH-type transcriptional regulator YdcR
120058	140469	20411	IslandPath-DIMOB		LCN16_00122	136975	137547	1	hypothetical protein
120058	140469	20411	IslandPath-DIMOB	yiaW_1	LCN16_00123	137708	138031	1	Inner membrane protein YiaW
120058	140469	20411	IslandPath-DIMOB	yibH	LCN16_00124	138037	139173	1	Inner membrane protein YibH
120058	140469	20411	IslandPath-DIMOB	esaR	LCN16_00125	139738	140469	1	Transcriptional activator protein EsaR
365100	371737	6637	SIGI-HMM		LCN16_00325	365100	365348	1	hypothetical protein

365100	371737	6637	SIGI-HMM	prnA	LCN16_00326	366011	367621	1	Flavin-dependent tryptophan halogenase PrnA
365100	371737	6637	SIGI-HMM	prnB	LCN16_00327	367621	368703	1	Monodechloroaminopyrrolnitrin synthase PrnB
365100	371737	6637	SIGI-HMM	soxA	LCN16_00328	368748	370451	1	Monomeric sarcosine oxidase
365100	371737	6637	SIGI-HMM	prnD	LCN16_00329	370473	371567	1	Aminopyrrolnitrin oxygenase PrnD
365100	371737	6637	SIGI-HMM	rutF_1	LCN16_00330	371567	371737	1	FMN reductase (NADH) RutF
365100	371737	6637	SIGI-HMM	nhaS3	LCN16_00331	371734	372978	1	High-affinity Na(+)/H(+) antiporter NhaS3
444162	448904	4742	IslandPath-DIMOB	argR_1	LCN16_00405	444568	445038	-1	Arginine repressor
444162	448904	4742	IslandPath-DIMOB	mdh	LCN16_00406	445504	446442	1	Malate dehydrogenase
444162	448904	4742	IslandPath-DIMOB		LCN16_00407	446511	446771	-1	DNA-binding transcriptional regulator Nlp
444162	448904	4742	IslandPath-DIMOB		LCN16_00408	446953	447303	1	Mu DNA-binding domain protein
444162	448904	4742	IslandPath-DIMOB	ispB	LCN16_00409	447352	448323	-1	Octaprenyl-diphosphate synthase
444162	448904	4742	IslandPath-DIMOB	rplU	LCN16_00410	448593	448904	1	50S ribosomal protein L21
547609	559681	5522	SIGI-HMM		LCN16_00509	554159	556597	1	hypothetical protein
547609	559681	5522	SIGI-HMM		LCN16_00510	557333	557602	1	hypothetical protein
547609	559681	5522	SIGI-HMM		LCN16_00511	557714	558046	-1	hypothetical protein
547609	559681	5522	SIGI-HMM		LCN16_00512	558067	558300	-1	hypothetical protein
547609	559681	5522	SIGI-HMM		LCN16_00513	558776	559681	1	hypothetical protein
547609	560881	13272	IslandPath-DIMOB	holC	LCN16_00503	547623	548075	-1	DNA polymerase III subunit chi
547609	560881	13272	IslandPath-DIMOB	pepA_1	LCN16_00504	548271	549782	-1	Cytosol aminopeptidase
547609	560881	13272	IslandPath-DIMOB	lptF	LCN16_00505	550063	551157	1	Lipopolysaccharide export system permease protein LptF
547609	560881	13272	IslandPath-DIMOB	lptG	LCN16_00506	551157	552227	1	Lipopolysaccharide export system permease protein LptG
547609	560881	13272	IslandPath-DIMOB	intA_1	LCN16_00508	552798	554060	1	Prophage CP4-57 integrase
547609	560881	13272	IslandPath-DIMOB		LCN16_00509	554159	556597	1	hypothetical protein

554159	560881	13272	IslandPath-DIMOB		LCN16_00510	557333	557602	1	hypothetical protein
554159	560881	13272	IslandPath-DIMOB		LCN16_00511	557714	558046	-1	hypothetical protein
554159	560881	13272	IslandPath-DIMOB		LCN16_00512	558067	558300	-1	hypothetical protein
554159	560881	13272	IslandPath-DIMOB		LCN16_00513	558776	559681	1	hypothetical protein
554159	560881	13272	IslandPath-DIMOB	cynT_1	LCN16_00514	560017	560652	1	Carbonic anhydrase 1
1651616	1663703	12087	SIGI-HMM		LCN16_01543	1651616	1652656	1	Polysaccharide biosynthesis/export protein
1651616	1663703	12087	SIGI-HMM	wzb	LCN16_01544	1652661	1653095	1	Low molecular weight protein-tyrosine-phosphatase wzb
1651616	1663703	12087	SIGI-HMM	wzc_1	LCN16_01545	1653108	1655279	1	Tyrosine-protein kinase wzc
1651616	1663703	12087	SIGI-HMM		LCN16_01546	1655443	1656564	1	Glycosyl transferases group 1
1651616	1663703	12087	SIGI-HMM		LCN16_01547	1656642	1658060	1	hypothetical protein
1651616	1663703	12087	SIGI-HMM	mshA	LCN16_01548	1658057	1659115	1	D-inositol 3-phosphate glycosyltransferase
1651616	1663703	12087	SIGI-HMM		LCN16_01549	1659173	1659904	1	hypothetical protein
1651616	1663703	12087	SIGI-HMM	wcaJ	LCN16_01550	1660481	1661914	1	UDP-glucose:undecaprenyl-phosphate glucose-1-phosphate transferase
1651616	1663703	12087	SIGI-HMM		LCN16_01551	1662096	1663703	1	hypothetical protein
1672845	1677779	4934	SIGI-HMM	rfbX	LCN16_01556	1672845	1674080	1	Putative O-antigen transporter
1672845	1677779	4934	SIGI-HMM	glf	LCN16_01557	1674080	1675177	1	UDP-galactopyranose mutase
1672845	1677779	4934	SIGI-HMM		LCN16_01558	1675186	1676169	1	hypothetical protein
1672845	1677779	4934	SIGI-HMM	manC1	LCN16_01559	1676355	1677779	1	Mannose-1-phosphate guanylyltransferase 1
1682972	1695141	12169	SIGI-HMM	rmlD_1	LCN16_01565	1682972	1683835	1	dTDP-4-dehydrorhamnose reductase
1682972	1695141	12169	SIGI-HMM		LCN16_01566	1683964	1684827	1	hypothetical protein
1682972	1695141	12169	SIGI-HMM	rfbC	LCN16_01567	1685390	1685923	1	dTDP-4-dehydrorhamnose 3,5-epimerase
1682972	1695141	12169	SIGI-HMM	rmlD_2	LCN16_01568	1686031	1686906	1	dTDP-4-dehydrorhamnose reductase
1682972	1695141	12169	SIGI-HMM	tagG	LCN16_01569	1686928	1687716	1	Teichoic acid translocation permease protein TagG
1682972	1695141	12169	SIGI-HMM	tagH	LCN16_01570	1687706	1689097	1	Teichoic acids export ATP-binding protein TagH
1682972	1695141	12169	SIGI-HMM		LCN16_01571	1689090	1693106	1	?-D-glucose-1-phosphatase

1682972	1695141	12169	SIGI-HMM		LCN16_01572	1693169	1694095	1	putative glycosyl transferase
1682972	1695141	12169	SIGI-HMM	wbbL	LCN16_01573	1694110	1694928	1	N-acetylglucosaminyl-diphospho-decaprenol L-rhamnosyltransferase
1682972	1695141	12169	SIGI-HMM		LCN16_01574	1694998	1695141	1	hypothetical protein
1891045	1897317	6272	SIGI-HMM	xerD_1	LCN16_01752	1891045	1892208	-1	Tyrosine recombinase XerD
1891045	1897317	6272	SIGI-HMM		LCN16_01753	1892590	1893258	-1	hypothetical protein
1891045	1897317	6272	SIGI-HMM		LCN16_01754	1893255	1893437	-1	hypothetical protein
1891045	1897317	6272	SIGI-HMM		LCN16_01755	1893453	1893842	-1	hypothetical protein
1891045	1897317	6272	SIGI-HMM		LCN16_01756	1894301	1894558	-1	hypothetical protein
1891045	1897317	6272	SIGI-HMM		LCN16_01757	1894617	1894982	-1	hypothetical protein
1891045	1897317	6272	SIGI-HMM		LCN16_01758	1894979	1895158	-1	hypothetical protein
1891045	1897317	6272	SIGI-HMM		LCN16_01759	1895161	1895565	-1	hypothetical protein
1891045	1897317	6272	SIGI-HMM		LCN16_01760	1895565	1895774	-1	hypothetical protein
1891045	1897317	6272	SIGI-HMM		LCN16_01761	1895771	1896199	-1	HNH endonuclease
1891045	1897317	6272	SIGI-HMM		LCN16_01762	1896192	1896707	-1	hypothetical protein
1891045	1897317	6272	SIGI-HMM		LCN16_01763	1896694	1897317	-1	hypothetical protein
1891045	1897317	6272	SIGI-HMM		LCN16_01764	1897314	1897808	-1	hypothetical protein
1893258	1918661	25403	IslandPath-DIMOB		LCN16_01754	1893255	1893437	-1	hypothetical protein
1893258	1918661	25403	IslandPath-DIMOB		LCN16_01755	1893453	1893842	-1	hypothetical protein
1893258	1918661	25403	IslandPath-DIMOB		LCN16_01756	1894301	1894558	-1	hypothetical protein
1893258	1918661	25403	IslandPath-DIMOB		LCN16_01757	1894617	1894982	-1	hypothetical protein
1893258	1918661	25403	IslandPath-DIMOB		LCN16_01758	1894979	1895158	-1	hypothetical protein
1893258	1918661	25403	IslandPath-DIMOB		LCN16_01759	1895161	1895565	-1	hypothetical protein
1893258	1918661	25403	IslandPath-DIMOB		LCN16_01760	1895565	1895774	-1	hypothetical protein
1893258	1918661	25403	IslandPath-DIMOB		LCN16_01761	1895771	1896199	-1	HNH endonuclease

1893258	1918661	25403	IslandPath-DIMOB		LCN16_01762	1896192	1896707	-1	hypothetical protein
1893258	1918661	25403	IslandPath-DIMOB		LCN16_01763	1896694	1897317	-1	hypothetical protein
1893258	1918661	25403	IslandPath-DIMOB		LCN16_01764	1897314	1897808	-1	hypothetical protein
1893258	1918661	25403	IslandPath-DIMOB		LCN16_01765	1897811	1897954	-1	hypothetical protein
1893258	1918661	25403	IslandPath-DIMOB		LCN16_01766	1897948	1898166	-1	hypothetical protein
1893258	1918661	25403	IslandPath-DIMOB		LCN16_01767	1898447	1898545	-1	hypothetical protein
1893258	1918661	25403	IslandPath-DIMOB		LCN16_01768	1898548	1898724	-1	hypothetical protein
1893258	1918661	25403	IslandPath-DIMOB		LCN16_01769	1898761	1899099	-1	hypothetical protein
1893258	1918661	25403	IslandPath-DIMOB		LCN16_01770	1899590	1899733	1	hypothetical protein
1893258	1918661	25403	IslandPath-DIMOB		LCN16_01771	1899774	1900172	-1	hypothetical protein
1893258	1918661	25403	IslandPath-DIMOB		LCN16_01772	1900169	1900834	-1	hypothetical protein
1893258	1918661	25403	IslandPath-DIMOB		LCN16_01773	1901245	1901889	-1	putative HTH-type transcriptional regulator
1893258	1918661	25403	IslandPath-DIMOB		LCN16_01774	1901981	1902208	1	hypothetical protein
1893258	1918661	25403	IslandPath-DIMOB		LCN16_01775	1902224	1902550	1	Bacteriophage CII protein
1893258	1918661	25403	IslandPath-DIMOB		LCN16_01776	1902834	1903598	1	Phage antirepressor protein KilAC domain protein
1893258	1918661	25403	IslandPath-DIMOB		LCN16_01777	1903601	1903777	1	hypothetical protein
1893258	1918661	25403	IslandPath-DIMOB		LCN16_01778	1903774	1904796	1	hypothetical protein
1893258	1918661	25403	IslandPath-DIMOB		LCN16_01779	1904793	1905764	1	hypothetical protein
1893258	1918661	25403	IslandPath-DIMOB		LCN16_01780	1906115	1906513	1	Phage antitermination protein Q
1893258	1918661	25403	IslandPath-DIMOB		LCN16_01781	1906767	1906949	1	hypothetical protein
1893258	1918661	25403	IslandPath-DIMOB		LCN16_01782	1906993	1907625	1	hypothetical protein

1893258	1918661	25403	IslandPath-DIMOB		LCN16_01783	1907890	1908237	1	hypothetical protein
1893258	1918661	25403	IslandPath-DIMOB		LCN16_01784	1908508	1908861	1	hypothetical protein
1893258	1918661	25403	IslandPath-DIMOB		LCN16_01785	1908953	1909189	1	Lysis protein S
1893258	1918661	25403	IslandPath-DIMOB	rrrD_1	LCN16_01786	1909192	1909677	1	Lysozyme RrrD
1893258	1918661	25403	IslandPath-DIMOB		LCN16_01787	1909674	1910048	1	hypothetical protein
1893258	1918661	25403	IslandPath-DIMOB	holE_1	LCN16_01788	1910641	1910895	1	DNA polymerase III subunit theta
1893258	1918661	25403	IslandPath-DIMOB		LCN16_01789	1910859	1910975	-1	hypothetical protein
1893258	1918661	25403	IslandPath-DIMOB		LCN16_01790	1911048	1911167	1	hypothetical protein
1893258	1918661	25403	IslandPath-DIMOB		LCN16_01791	1911248	1911658	1	hypothetical protein
1893258	1918661	25403	IslandPath-DIMOB		LCN16_01792	1911758	1912435	1	hypothetical protein
1893258	1918661	25403	IslandPath-DIMOB		LCN16_01793	1912373	1912675	-1	hypothetical protein
1893258	1918661	25403	IslandPath-DIMOB		LCN16_01794	1912820	1913035	-1	hypothetical protein
1893258	1918661	25403	IslandPath-DIMOB		LCN16_01795	1913062	1913592	1	Terminase small subunit
1893258	1918661	25403	IslandPath-DIMOB		LCN16_01796	1913589	1914848	1	Phage terminase large subunit
1893258	1918661	25403	IslandPath-DIMOB		LCN16_01797	1914904	1915098	1	hypothetical protein
1893258	1918661	25403	IslandPath-DIMOB		LCN16_01798	1915154	1916482	1	hypothetical protein
1893258	1918661	25403	IslandPath-DIMOB		LCN16_01799	1916466	1917392	1	Phage Mu protein F like protein
1893258	1918661	25403	IslandPath-DIMOB		LCN16_01800	1917396	1918661	1	hypothetical protein
1924033	1931063	7030	SIGI-HMM		LCN16_01810	1924033	1924242	1	hypothetical protein
1924033	1931063	7030	SIGI-HMM		LCN16_01811	1924246	1925076	1	hypothetical protein
1924033	1931063	7030	SIGI-HMM		LCN16_01812	1925224	1925373	1	hypothetical protein
1924033	1931063	7030	SIGI-HMM		LCN16_01813	1925445	1926350	-1	hypothetical protein

1924033	1931063	7030	SIGI-HMM		LCN16_01814	1926511	1926795	1	Arc-like DNA binding domain protein
1924033	1931063	7030	SIGI-HMM		LCN16_01815	1926905	1927186	1	hypothetical protein
1924033	1931063	7030	SIGI-HMM		LCN16_01816	1927255	1930437	1	hypothetical protein
1924033	1931063	7030	SIGI-HMM		LCN16_01817	1930440	1931063	1	hypothetical protein
2150029	2158352	8323	SIGI-HMM		LCN16_02045	2150029	2150928	1	hypothetical protein
2150029	2158352	8323	SIGI-HMM		LCN16_02046	2151182	2152684	1	hypothetical protein
2150029	2158352	8323	SIGI-HMM		LCN16_02047	2153053	2153484	1	hypothetical protein
2150029	2158352	8323	SIGI-HMM		LCN16_02048	2153539	2154168	1	hypothetical protein
2150029	2158352	8323	SIGI-HMM		LCN16_02049	2154334	2155458	-1	hypothetical protein
2150029	2158352	8323	SIGI-HMM		LCN16_02050	2155439	2155684	-1	Excisionase-like protein
2150029	2158352	8323	SIGI-HMM		LCN16_02051	2155684	2156181	-1	hypothetical protein
2150029	2158352	8323	SIGI-HMM		LCN16_02052	2156561	2157049	1	hypothetical protein
2150029	2158352	8323	SIGI-HMM		LCN16_02053	2157495	2157785	-1	hypothetical protein
2150029	2158352	8323	SIGI-HMM		LCN16_02054	2158068	2158352	1	Acetyltransferase (GNAT) family protein
2787314	2795303	7989	SIGI-HMM		LCN16_02636	2787314	2788036	1	ChaC-like protein
2787314	2795303	7989	SIGI-HMM		LCN16_02637	2788140	2788604	1	SnoaL-like polyketide cyclase
2787314	2795303	7989	SIGI-HMM	ada_1	LCN16_02638	2788813	2789394	1	Bifunctional transcriptional activator/DNA repair enzyme Ada
2787314	2795303	7989	SIGI-HMM		LCN16_02639	2789391	2790110	1	hypothetical protein
2787314	2795303	7989	SIGI-HMM	alkB_1	LCN16_02640	2790113	2790763	1	Alpha-ketoglutarate-dependent dioxygenase AlkB
2787314	2795303	7989	SIGI-HMM		LCN16_02641	2790839	2791033	1	hypothetical protein
2787314	2795303	7989	SIGI-HMM	alkA_1	LCN16_02642	2791079	2791729	1	DNA-3-methyladenine glycosylase
2787314	2795303	7989	SIGI-HMM	ada_2	LCN16_02643	2791808	2792878	1	Bifunctional transcriptional activator/DNA repair enzyme Ada
2787314	2795303	7989	SIGI-HMM		LCN16_02644	2792893	2793585	1	hypothetical protein
2787314	2795303	7989	SIGI-HMM		LCN16_02645	2793602	2793907	1	hypothetical protein
2787314	2795303	7989	SIGI-HMM		LCN16_02646	2793904	2794665	1	hypothetical protein
2787314	2795303	7989	SIGI-HMM	lexA_1	LCN16_02647	2794881	2795303	1	LexA repressor

3366019	3371308	5289	SIGI-HMM		LCN16_03205	3366019	3366234	1	hypothetical protein
3366019	3371308	5289	SIGI-HMM	dnaJ_2	LCN16_03206	3366519	3368021	-1	Chaperone protein DnaJ
3366019	3371308	5289	SIGI-HMM		LCN16_03207	3369443	3370039	-1	hypothetical protein
3366019	3371308	5289	SIGI-HMM		LCN16_03208	3370125	3370802	1	HTH domain protein
3366019	3371308	5289	SIGI-HMM		LCN16_03209	3370862	3371308	-1	universal stress protein UspC
3391931	3403430	11499	SIGI-HMM		LCN16_03227	3391931	3392473	1	hypothetical protein
3391931	3403430	11499	SIGI-HMM	dmlR_33	LCN16_03228	3392531	3393448	-1	HTH-type transcriptional regulator DmlR
3391931	3403430	11499	SIGI-HMM	rpoD_1	LCN16_03229	3395608	3397203	-1	RNA polymerase sigma factor RpoD
3391931	3403430	11499	SIGI-HMM		LCN16_03230	3397805	3398731	1	hypothetical protein
3391931	3403430	11499	SIGI-HMM	acr1	LCN16_03231	3398893	3399636	-1	Fatty acyl-CoA reductase
3391931	3403430	11499	SIGI-HMM		LCN16_03232	3399832	3400293	-1	hypothetical protein
3391931	3403430	11499	SIGI-HMM	lexA_2	LCN16_03233	3400689	3401081	-1	LexA repressor
3391931	3403430	11499	SIGI-HMM		LCN16_03234	3401214	3401630	-1	hypothetical protein
3391931	3403430	11499	SIGI-HMM	msbA_3	LCN16_03235	3401676	3403430	-1	Lipid A export ATP-binding/permease protein MsbA
3977324	4016722	9531	SIGI-HMM		LCN16_03790	4007191	4007451	-1	hypothetical protein
3977324	4016722	9531	SIGI-HMM		LCN16_03791	4007608	4008171	1	Bacteriophage CI repressor helix-turn-helix domain protein
3977324	4016722	9531	SIGI-HMM	xerD_2	LCN16_03792	4008175	4009242	1	Tyrosine recombinase XerD
3977324	4016722	9531	SIGI-HMM		LCN16_03793	4009314	4009529	-1	hypothetical protein
3977324	4016722	9531	SIGI-HMM	intA_2	LCN16_03794	4009694	4010416	1	Prophage CP4-57 integrase
3977324	4016722	39398	IslandPath-DIMOB	bamE	LCN16_03751	3977324	3977662	1	Outer membrane protein assembly factor BamE precursor
3977324	4016722	39398	IslandPath-DIMOB	pasI	LCN16_03752	3977777	3978061	-1	Persistence and stress-resistance antitoxin PasI
3977324	4016722	39398	IslandPath-DIMOB	ratA	LCN16_03753	3978042	3978488	-1	Ribosome association toxin RatA
3977324	4016722	39398	IslandPath-DIMOB	smpB	LCN16_03754	3978650	3979132	1	SsrA-binding protein
3977324	4016722	39398	IslandPath-DIMOB		LCN16_03756	3979628	3979867	-1	hypothetical protein
3977324	4016722	39398	IslandPath-DIMOB		LCN16_03757	3980574	3982229	-1	hypothetical protein

3977324	4016722	39398	IslandPath-DIMOB		LCN16_03758	3982226	3982786	-1	hypothetical protein
3977324	4016722	39398	IslandPath-DIMOB		LCN16_03759	3982761	3983483	-1	hypothetical protein
3977324	4016722	39398	IslandPath-DIMOB		LCN16_03760	3983473	3984021	-1	Caudovirales tail fiber assembly protein
3977324	4016722	39398	IslandPath-DIMOB		LCN16_03761	3984025	3987141	-1	Tail fiber protein
3977324	4016722	39398	IslandPath-DIMOB		LCN16_03762	3987147	3987752	-1	hypothetical protein
3977324	4016722	39398	IslandPath-DIMOB		LCN16_03763	3987745	3988929	-1	Baseplate J-like protein
3977324	4016722	39398	IslandPath-DIMOB		LCN16_03764	3988907	3989254	-1	hypothetical protein
3977324	4016722	39398	IslandPath-DIMOB		LCN16_03765	3989254	3991785	-1	Phage-related minor tail protein
3977324	4016722	39398	IslandPath-DIMOB		LCN16_03766	3991973	3992242	-1	hypothetical protein
3977324	4016722	39398	IslandPath-DIMOB		LCN16_03767	3992390	3992734	-1	hypothetical protein
3977324	4016722	39398	IslandPath-DIMOB		LCN16_03768	3992734	3993075	-1	hypothetical protein
3977324	4016722	39398	IslandPath-DIMOB		LCN16_03769	3993062	3993364	-1	Phage holin family 2
3977324	4016722	39398	IslandPath-DIMOB		LCN16_03770	3993374	3993829	-1	hypothetical protein
3977324	4016722	39398	IslandPath-DIMOB		LCN16_03771	3993826	3994950	-1	hypothetical protein
3977324	4016722	39398	IslandPath-DIMOB		LCN16_03772	3994947	3995657	-1	Phage virion morphogenesis family protein
3977324	4016722	39398	IslandPath-DIMOB		LCN16_03773	3995654	3996157	-1	P2 phage tail completion protein R (GpR)
3977324	4016722	39398	IslandPath-DIMOB		LCN16_03774	3996154	3996606	-1	Phage head completion protein (GPL)
3977324	4016722	39398	IslandPath-DIMOB		LCN16_03775	3996706	3997410	-1	Phage small terminase subunit
3977324	4016722	39398	IslandPath-DIMOB		LCN16_03776	3997417	3998433	-1	Phage major capsid protein, P2 family
3977324	4016722	39398	IslandPath-DIMOB		LCN16_03777	3998482	3999321	-1	Phage capsid scaffolding protein (GPO) serine peptidase
3977324	4016722	39398	IslandPath-DIMOB		LCN16_03778	3999631	4001268	1	Terminase-like family protein

3977324	4016722	39398	IslandPath-DIMOB		LCN16_03779	4001265	4002314	1	Phage portal protein
3977324	4016722	39398	IslandPath-DIMOB		LCN16_03780	4002365	4002637	1	Ogr/Delta-like zinc finger
3977324	4016722	39398	IslandPath-DIMOB		LCN16_03781	4002608	4002826	-1	hypothetical protein
3977324	4016722	39398	IslandPath-DIMOB		LCN16_03782	4002917	4004926	-1	Bacteriophage replication gene A protein (GPA)
3977324	4016722	39398	IslandPath-DIMOB		LCN16_03783	4004920	4005189	-1	hypothetical protein
3977324	4016722	39398	IslandPath-DIMOB		LCN16_03784	4005198	4005305	-1	hypothetical protein
3977324	4016722	39398	IslandPath-DIMOB		LCN16_03785	4005287	4005526	-1	hypothetical protein
3977324	4016722	39398	IslandPath-DIMOB		LCN16_03786	4005604	4006011	-1	hypothetical protein
3977324	4016722	39398	IslandPath-DIMOB		LCN16_03787	4006014	4006433	-1	hypothetical protein
3977324	4016722	39398	IslandPath-DIMOB		LCN16_03788	4006436	4006639	-1	hypothetical protein
3977324	4016722	39398	IslandPath-DIMOB		LCN16_03789	4006649	4007158	-1	Phage regulatory protein CII (CP76)
3977324	4016722	39398	IslandPath-DIMOB		LCN16_03790	4007191	4007451	-1	hypothetical protein
3977324	4016722	39398	IslandPath-DIMOB		LCN16_03791	4007608	4008171	1	Bacteriophage CI repressor helix-turn-helix domain protein
3977324	4016722	39398	IslandPath-DIMOB	xerD_2	LCN16_03792	4008175	4009242	1	Tyrosine recombinase XerD
3977324	4016722	39398	IslandPath-DIMOB		LCN16_03793	4009314	4009529	-1	hypothetical protein
3977324	4016722	39398	IslandPath-DIMOB	intA_2	LCN16_03794	4009694	4010416	1	Prophage CP4-57 integrase
3977324	4016722	39398	IslandPath-DIMOB		LCN16_03795	4010786	4013506	1	hypothetical protein
4007191	4016722	9531	SIGI-HMM		LCN16_03795	4010786	4013506	1	hypothetical protein
4007191	4016722	9531	SIGI-HMM		LCN16_03796	4013639	4013845	1	hypothetical protein
4007191	4016722	9531	SIGI-HMM		LCN16_03797	4014198	4014545	-1	hypothetical protein
4007191	4016722	9531	SIGI-HMM		LCN16_03798	4014738	4014872	-1	hypothetical protein
4007191	4016722	9531	SIGI-HMM		LCN16_03799	4014918	4015613	1	hypothetical protein

4007191	4016722	9531	SIGI-HMM	zinT	LCN16_03800	4016072	4016722	1	Metal-binding protein ZinT precursor
4007191	4016722	39398	IslandPath-DIMOB		LCN16_03796	4013639	4013845	1	hypothetical protein
4007191	4016722	39398	IslandPath-DIMOB		LCN16_03797	4014198	4014545	-1	hypothetical protein
4007191	4016722	39398	IslandPath-DIMOB		LCN16_03798	4014738	4014872	-1	hypothetical protein
4007191	4016722	39398	IslandPath-DIMOB		LCN16_03799	4014918	4015613	1	hypothetical protein
4007191	4016722	39398	IslandPath-DIMOB	zinT	LCN16_03800	4016072	4016722	1	Metal-binding protein ZinT precursor
4208425	4214408	5983	SIGI-HMM	hisN	LCN16_03983	4208425	4209237	-1	Histidinol-phosphatase
4208425	4214408	5983	SIGI-HMM		LCN16_03984	4209552	4210094	-1	NUDIX domain protein
4208425	4214408	5983	SIGI-HMM	kanB	LCN16_03985	4210250	4211473	-1	2'-deamino-2'-hydroxyneamine transaminase
4208425	4214408	5983	SIGI-HMM	asnO	LCN16_03986	4211492	4212532	-1	L-asparagine oxygenase
4208425	4214408	5983	SIGI-HMM	mdtL_2	LCN16_03987	4213239	4214408	1	Multidrug resistance protein MdtL
4941042	4945478	4436	SIGI-HMM		LCN16_04651	4941042	4941200	-1	BetR domain protein
4941042	4945478	4436	SIGI-HMM		LCN16_04652	4943169	4943702	1	hypothetical protein
4941042	4945478	4436	SIGI-HMM		LCN16_04653	4944852	4945478	1	hypothetical protein
4941042	4945478	4436	SIGI-HMM		LCN16_04654	4945401	4948502	-1	Extracellular serine protease precursor
