# Supplementary material for: The genome and genetics of a high oxidative stress tolerant Serratia sp. LCN16 isolated from the plant parasitic nematode Bursaphelenchus xylophilus
Source: BMC Genomics. 2016 Apr 23;17:301. doi: 10.1186/s12864-016-2626-1 (PMC4841953; doi:10.1186/s12864-016-2626-1)
Supplement: Additional file 4: Table S3. — List of genes predicted to be involved in a plant-associated life-style of Serratia sp. LCN16. (PDF 113 kb) [file 12864_2016_2626_MOESM4_ESM.pdf]

Function	Gene reference Name	Gene reference Number	KEGG annotation	Description
ABC Transporters				
Iron (III) transporter	*afuA*	LCN16_02557	K02012	substrate-binding protein
		LCN16_03143		
		LCN16_04047		
	afuB	LCN16_02558	K02011	permease protein
		LCN16_03144		
		LCN16_04046		
	afuC	LCN16_02559	K02010	ATP-binding protein
		LCN16_03145		
		LCN16_04045		
Putrescine transporter	*potF*	LCN16_01614	K11073	substrate-binding protein
	potG	LCN16_01615	K11076	ATP-binding protein
	potH	LCN16_01616	K11075	permease protein
	potI	LCN16_01617	K11074	permease protein
Spermidine/putrescine		LCN16_03709	K02055	substrate-binding protein
		LCN16_04432	K02055	
		LCN16_03706	K02053	permease protein
		LCN16_04429	K02053	
		LCN16_03707	K02054	
		LCN16_04430	K02054	
		LCN16_03708	K02052	ATP-binding protein
		LCN16_04428	K02052	
Iron complex transporter		LCN16_00483	K02016	substrate-binding protein
		LCN16_00935		
		LCN16_01231		
		LCN16_01361		
		LCN16_02183		
		LCN16_03258		
		LCN16_03495		
		LCN16_04075		
		LCN16_00482	K02015	permease protein
		LCN16_01233		
		LCN16_01317		
		LCN16_02182		
		LCN16_03260		

		LCN16_03497		
		LCN16_03498		
		LCN16_04074		
		LCN16_00484	K02013	ATP-binding protein
		LCN16_01234		
		LCN16_01318		
		LCN16_02181		
		LCN16_03259		
		LCN16_03499		
		LCN16_04076		
Manganese/iron	*sitA*	LCN16_02147	K11604	substrate-binding protein
	sitC	LCN16_02145	K11605	permease protein
	sitD	LCN16_02144	K11606	
	sitB	LCN16_02146	K11607	ATP-binding protein
Electrochemical potential-driven	*efeU*	LCN16_02958	K07243	high-affinity iron transporter
	efeO	LCN16_02957	K07224	iron uptake system
	efeB	LCN16_02956	K16301	deferrochelatase/peroxidase
Others	*feoA*	LCN16_04566	K04758	ferrous iron transport protein
	feoB	LCN16_04567	K04759	
	feoC	LCN16_04568	K07490	
Motility and Chemotaxis				
	cheA	LCN16_03016	K03407	two-component system, chemotaxis family, sensorkinase CheA [EC:2.7.13.3]
	cheW	LCN16_03015	K03408	purine-binding chemotaxis protein CheW
	cheR	LCN16_03012	K00575	chemotaxis protein methyltransferase CheR [EC:2.1.1.80]
	cheB	LCN16_03011	K03412	two-componentsystem,chemotaxisfamily,responseregulatorCheB[EC:3.1.1.61]
	cheY	LCN16_03010	K03413	two-componentsystem,chemotaxisfamily,responseregulatorCheY
	cheZ	LCN16_03009	K03414	chemotaxisproteinCheZ
	mcp	LCN16_02979	K03406	Methyl-accepting chemotaxis protein I
	tsr	LCN16_03014	K05874	tsr; methyl-accepting chemotaxis protein I, serine sensor receptor
	tar	LCN16_00083	K05877	tap; methyl-accepting chemotaxis protein IV, peptide sensor receptor
	tap	LCN16_03013	K05877	tap; methyl-accepting chemotaxis protein IV, peptide sensor receptor
Adhesion				
	fhaB	LCN16_04167	K1525	filamentous hemaglutinnin
	fhaB	LCN16_04346	K1526	filamentous hemaglutinnin
Plant polymer degradation				
		LCN16_00161	K01179	endoglucanase [EC:3.2.1.4]

	uxaA	LCN16_04263	K01686	mannonate dehydratase
	uxaC	LCN16_04264	K00040	fructuronate reductase
Plant growth promotion				
Nitrogen metabolism	*glnL*	LCN16_04783		two-component regulatory system
	glnG	LCN16_04784		two-component regulatory system
	narX	LCN16_02937		nitrogen respiration
	narL	LCN16_02938		nitrogen respiration
	narQ	LCN16_03562		nitrate respiration
	narP	LCN16_03563		nitrate respiration
Siderophore	*entC*	LCN16_03494	K02364	enterobactin synthetase component F
	entE	LCN16_03493	K02363	2,3-dihydro-2,3-dihydroxybenzoate-AMP ligase
	entA	LCN16_03491	K02361	2,3-dihydro-2,3-dihydroxybenzoate dehydrogenase
	entF	LCN16_00890	K02364	enterobactin synthetase component F
		LCN16_03500		
	exbD	LCN16_00528	K03558	Biopolymer transport protein ExbD
		LCN16_04176	K03559	Biopolymer transport protein ExbD
	exbB	LCN16_00531	K03561	Biopolymer transport protein ExbB
		LCN16_04177	K03562	Biopolymer transport protein ExbB
	bfr	LCN16_04493	K03594	bacterioferritin
	menF	LCN16_03345	K02552	menaquinone-specific isochorismate synthase
	pchB	LCN16_03570	K04782	isochorismate pyruvate lyase
IAA	*ipdC*	LCN16_00911	K04103	indole-3-pyruvate decarboxylase
		LCN16_03478		
	budA	LCN16_03505	K01575	Acetolactate decarboxylase
	budB	LCN16_03506	K01652	Acetolactate synthase, catabolic
	budC	LCN16_02073	K18009	(S,S)-butanediol dehydrogenase (acetoin reductase)
Aromatic Degradation	*aaeA*	LCN16_04337	K15548	p-hydroxybenzoic acid efflux pump
	aaeB	LCN16_04336	K03468	p-hydroxybenzoic acid efflux pump
	catA	LCN16_03045	K03381	catechol 1,2-dioxygenase
	catB	LCN16_03043	K01856	muconate D-isomerase
	catC	LCN16_03044	K03464	muconolactone D-isomerase
	benA	LCN16_03046	K05549	benzoate/toluate 1,2-oxygenase alpha subunit
	benB	LCN16_03047	K05550	benzoate/toluate 1,2-oxygenase beta subunit
	benC	LCN16_03048	K05784	benzoate/toluate 1,2-oxygenase reductase subunit
	benD	LCN16_03049	K05783	dihydroxycyclohexadiene carboxylate dehydrogenase
	pcaD	LCN16_03042	K01055	3-oxoadipate enol-lactonase
	pcaG	LCN16_02516	K00448	protocatechuate 3,4-dioxygenase alpha subunit
	pcaH	LCN16_02515	K00449	protocatechuate 3,4-dioxygenase beta subunit
	pcaB	LCN16_02519	K01857	3-carboxy-cis, cis-muconate cycloisomerase

	pcaC	LCN16_01415	K01607	4-carboxymuconolactone decarboxylase
	pcaC	LCN16_02518	K01607	4-carboxymuconolactone decarboxylase
	pobA	LCN16_02520	K00481	p-hydroxybenzoate 3-monoxygenase
	hpaB	LCN16_00566	K00483	4-hydroxyphenylacetate 3-monoxygenase
	hpaC	LCN16_00567	K00484	flavin reductase
	hpaD	LCN16_00560	K00455	3,4-dihydroxyphenylacetate 2,3-dioxygenase
	hpaE	LCN16_00559	K00151	5-carboxymethyl-2-hydroxymuconic-semialdehyde dehydrogenase
	hpaF	LCN16_00561	K01826	5-carboxymethyl-2-hydroxymuconate isomerase
	hpaG	LCN16_00557	K05921	2-hydroxyhepta-2,4-diene-1,7-dioate isomerase
	hapG	LCN16_00558	K05921	2-hydroxyhepta-2,4-diene-1,7-dioate isomerase
**Secretion system proteins**				
T1SS	*tolC*	LCN16_04212	K12340	outer membrane protein
	hasD	LCN16_01553	K12536	bacterial exporter for protease/lipase
	hasE	LCN16_01554	K12537	protease secretion system
	hasF	LCN16_01555	K12538	protease secretion system
T2SS	*gspS*	LCN16_03133	K02465	general secretion pathway protein S
T5SS	*ssp*	LCN16_04654	K12685	subtilase-type serine protease
	apeE	LCN16_00070	K12686	outer membrane lipase/esterase
Sec-dependent	*secA*	LCN16_00698	K03070	preprotein translocase subunit
	secB	LCN16_04731	K03071	preprotein translocase subunit
	secD	LCN16_00975	K03072	preprotein translocase subunit
	secE	LCN16_00287	K03073	preprotein translocase subunit
	secF	LCN16_00976	K03074	preprotein translocase subunit
	secG	LCN16_00423	K03075	preprotein translocase subunit
	secM	LCN16_00697	K13301	secretion monitor
	secY	LCN16_04471	K03076	preprotein translocase subunit
	ffh	LCN16_00786	K03106	
	ftsY	LCN16_00237	K03110	
	yajC	LCN16_00974	K03210	preprotein translocase subunit
	yidC	LCN16_00044	K03217	
Tat-independent	*tatA*	LCN16_00270	K03116	sec-independent protein translocase protein
	tatA	LCN16_01113	K03116	sec-independent protein translocase protein
	tatB	LCN16_00271	K03117	sec-independent protein translocase protein
	tatC	LCN16_00272	K03118	sec-independent protein translocase protein
**Antimicrobial Compounds**				
	hpmA	LCN16_04426	K11016	hemolysin
	hpmB	LCN16_04425	K11017	hemolysin activation/secretion protein
	hlyIII	LCN16_04002	K11068	hemolysin III
	plcC	LCN16_04159	K01114	phospholipase C

	tlh	LCN16_04200	K11018	thermostable hemolysin
		LCN16_00223	K01406	serralysin
		LCN16_02109		serralysin
		LCN16_03865		serralysin
	prnA	LCN16_00326		pyrrolnitrin
	prnB	LCN16_00327		
	prnD	LCN16_00329		
	kanB	LCN16_03985		Kanamycin biosynthesis
	mdtL	LCN16_03987		Chroramphenicol resistance
	ampC	LCN16_02298	K01467	beta-Lactamase classe C
	macA	LCN16_01639	K13888	macrolide-specific efflux system
		LCN16_01870		
		LCN16_03524		
	macB	LCN16_01640	K05685	macrolide transport system ATP-binding/permease
		LCN16_01869		
		LCN16_03525		
	emrA	LCN16_01268	K03543	multidrug efflux system
		LCN16_03848		
	acrA	LCN16_01039	K03585	
		LCN16_02497		
	acrB	LCN16_01038	K18138	
		LCN16_02496		
		LCN16_02547		
	marC	LCN16_02155	K05595	multiple antibiotic resistance
	hcnA	LCN16_01840		Hydrogen cyanide synthase subunit HcnC
	hcnB	LCN16_01841		Hydrogen cyanide synthase subunit HcnA
	hcnC	LCN16_01842		Hydrogen cyanide synthase subunit HcnB
Antifungal activity		LCN16_00148	K01183	chitinase [EC:3.2.1.14]
		LCN16_01150	K01184	chitinase [EC:3.2.1.14]
		LCN16_02767	K01185	chitinase [EC:3.2.1.14]
		LCN16_03549	K01186	chitinase [EC:3.2.1.14]
